# Application of single-cell RNA sequencing analysis of novel breast cancer phenotypes based on the activation of ferroptosis-related genes

**DOI:** 10.1007/s10142-023-01086-0

**Published:** 2023-05-22

**Authors:** Shuochuan Liu, Yajie Zhao, Jiao Zhang, Zhenzhen Liu

**Affiliations:** Department of Breast Disease, Henan Breast Cancer Center, Affiliated Cancer Hospital of Zhengzhou University & Henan Cancer Hospital, Dongming Road, Zhengzhou, 450008 Henan Province China

**Keywords:** Single-cell RNA sequencing, Ferroptosis, Breast cancer, Tumor microenvironment

## Abstract

Ferroptosis is distinct from classic apoptotic cell death characterized by the accumulation of reactive oxygen species (ROS) and lipid peroxides on the cell membrane. Increasing findings have demonstrated that ferroptosis plays an important role in cancer development, but the exploration of ferroptosis in breast cancer is limited. In our study, we aimed to establish a ferroptosis activation-related model based on the differentially expressed genes between a group exhibiting high ferroptosis activation and a group exhibiting low ferroptosis activation. By using machine learning to establish the model, we verified the accuracy and efficiency of our model in The Cancer Genome Atlas Breast Invasive Carcinoma (TCGA-BRCA) set and gene expression omnibus (GEO) dataset. Additionally, our research innovatively utilized single-cell RNA sequencing data to systematically reveal the microenvironment in the high and low FeAS groups, which demonstrated differences between the two groups from comprehensive aspects, including the activation condition of transcription factors, cell pseudotime features, cell communication, immune infiltration, chemotherapy efficiency, and potential drug resistance. In conclusion, different ferroptosis activation levels play a vital role in influencing the outcome of breast cancer patients and altering the tumor microenvironment in different molecular aspects. By analyzing differences in ferroptosis activation levels, our risk model is characterized by a good prognostic capacity in assessing the outcome of breast cancer patients, and the risk score can be used to prompt clinical treatment to prevent potential drug resistance. By identifying the different tumor microenvironment landscapes between the high- and low-risk groups, our risk model provides molecular insight into ferroptosis in breast cancer patients.

**Supplementary Information:**

The online version contains supplementary material available at 10.1007/s10142-023-01086-0.

## Introduction

Recent studies have revealed that breast cancer has become the most commonly diagnosed cancer in women worldwide and is characterized by the second highest mortality, followed by lung cancer (Starek-Swiechowicz et al. 2021). Breast cancer is classically divided into four subtypes according to immunohistology, but as a heterogeneous disease, novel subtypes of breast cancer can be further identified according to different genetic and metabolic landscapes (Perou et al. 2000; Cancer Genome Atlas Network 2012; Curtis et al. 2012; Jiang et al. 2019), which is helpful for individualized treatment and improving survival outcomes. Currently, even with the development of breast cancer treatment, breast cancer patients still face challenges, including recurrence or drug resistance (Burguin et al. 2021), among which the progression of drug resistance is a leading issue.

Ferroptosis, as a novel form of necrotic cell death, is characterized by oxidative modification through an iron-related mechanism (Conrad et al. 2016). It has also been identified as programmed cell death based on reactive oxygen species (ROS)-activated lipid peroxidation (Sui et al. 2022). Previous studies have revealed that ferroptosis is regulated in three main ways. First, the generation of ROS has been confirmed to regulate ferroptosis (Louandre et al. 2013). Second, it is influenced by the GPX4-catalyzed neutral reaction between glutathione (GSH) and ROS (Shi et al. 2021). Additionally, lipid peroxidation induced by the catalysis of arachidonate lipoxygenases (ALOXs) has been proven to influence ferroptosis (Jiang et al. 2021). With the increasing exploration of ferroptosis, many studies have determined its important roles in influencing cancer occurrence and progression. The death of cancer cells is triggered in pancreatic ductal adenocarcinoma (PDAC) by the ferroptosis inducer erastin (Gao et al. 2018). Moreover, ferroptosis has been indicated to improve cisplatin resistance in head and neck tumors (Shin et al. 2018). Studies have also indicated that ferroptosis is correlated with epithelial-to-mesenchymal transition (EMT) in breast cancer cells, which is associated with lipid metabolism (Feng and Kurokawa 2020). However, knowledge regarding the actual roles of ferroptosis in breast cancer is still limited, and the potential relationship between ferroptosis and breast cancer is worth exploring.

Single-cell RNA sequencing is a novel technique that plays important roles in identifying and characterizing cell types, cancer subtypes, disease states, and so on (Haque et al. 2017; Tanay and Regev 2017). With the development of computational methods, single-cell RNA sequencing enables the analysis of high-throughput sequencing data for different microenvironmental explorations of cell differentiation and gene regulation, especially during cancer progression, which is useful to provide insights into vital biological processes or identify key biomarkers (Wu et al. 2017).

Breast cancer is a malignant tumor with high heterogeneity, and the accurate classification of distinctive biological landscapes in breast cancer is valuable since it will help to predict the prognosis of patients and guide the use of personalized therapy in clinical practice. Therefore, in our current study, we established a ferroptosis activation-related risk score to predict the outcome of breast cancer patients and revealed the microenvironment in breast cancer based on the two subtypes of ferroptosis activation. We aimed to use the ferroptosis activation level to divide patients into high and low activation groups to further explore the differences between these two novel subtypes of breast cancer patients, who few other researchers have studied. However, ferroptosis has been found to be involved in many biological processes during cancer progression, and taking advantage of newly developed technologies is necessary to further explore the potential influence of ferroptosis on the tumor microenvironment in breast cancer. In our current study, we innovatively leveraged single-cell RNA sequencing data to further reveal the microenvironment in breast cancer based on our novel ferroptosis activation-related risk score model and proved the efficiency and accuracy of our model. Our study aimed to verify the ferroptosis activation-related risk score model, which included analyses of transcription factors, cell pseudotime features, cancer cell communication, clinical treatment, and drug resistance and provided a full landscape of the differences between individuals with high ferroptosis activation and low ferroptosis activation.

## Method

### Data collection

Our study utilized bulk sequencing data and single-cell RNA sequencing data. The bulk sequencing data consisted of a training set and validation set, which included the TCGA-BRCA set and GSE96058, respectively. The TCGA-BRCA dataset included transcriptome data, mutation data, copy number variant data, and clinical information that were downloaded from Xena (https://xenabrowser.net/datapages/). The GSE96058 dataset included transcriptome data and clinical information. The GSE96058 transcriptome dataset is a next-generation sequencing dataset that was downloaded using the R package GEO query (Davis and Meltzer 2007).

### Ferroptosis-related gene set

The ferroptosis-related genes were selected based on PMID: 28,985,560, PMID: 31,634,900, PMID: 31,634,899, and PMID: 31,105,042 (Stockwell et al. 2017; Bersuker et al. 2019; Doll et al. 2019; Hassannia et al. 2019). By combining the above four studies, we collected 60 ferroptosis-related genes with high confidence.

### Data preprocessing

The GSE176078 dataset included single-cell RNA sequencing data containing 100,064 single-cell transcript sequencing samples (https://www.ncbi.nlm.nih.gov/geo/download/?acc=GSE176078&form at = file). This dataset contained single-cell sequencing data obtained from 26 tumor samples, and the detailed sample collection method was based on PMID: 34,493,872 (Wu et al. 2021). The merge function of the Seurat R package (version 4.1.1) was used to integrate the samples. The newly merged dataset had 27,719 gene sequencing results containing 100,064 cells. Sequencing quality control was based on the following standards: unique molecular identifier (UMI) > 1000, 200 < single-cell gene count < 6000, and mitochondrial gene expression < 20% (UMI count). For batch correction, the harmony package was used (Korsunsky et al. 2019).

### Single-cell subtype annotation

Using the SingleR package, the annotation of single-cell subtypes was performed based on annotation information from the Human Primary Cell Atlas (Mabbott et al. 2013).

### Single-cell copy number variation profile

Single-cell copy number variation profiles were analyzed using CopyKAT (Gao et al. 2021) (id.type = “S,” ngene.chr = 5, win.size = 25, KS.cut = 0.1).

### Distinguishing malignant cells from nonmalignant cells

CopyKAT was used to cluster copy number variation in epithelial and normal cells. Clusters were divided into two categories: normal epithelial cells were defined as nonmalignant cells, and others were defined as malignant cells.

### Consensus clustering analysis

Based on the ferroptosis-related genes identified using the Consensus Cluster Plus package, samples from the TCGA-BRCA set were clustered. The GeoTcgaData package was used for transcripts per million (TPM) transformation. The clustering parameters were as follows: product-limit method, Euclidean distance, seed = 1, k.max = 5.

### Identification of differentially expressed genes

The count data from the TCGA-BRCA set were analyzed. Based on the gene expression ratio from cluster1 and cluster2 during the calculation of gene expression in cluster1 and gene expression in cluster2 and after performing log2FC calculations, we considered a ratio > 0.58 or ratio < 0.58 as indicators of differential expression. The screening threshold for differentially expressed genes was padj < 0.05 and |log2FoldChange|> 0.58.

### Identification of prognosis-related genes

According to the ratio of gene expression between cluster 1 and cluster 2, the differentially expressed genes were identified, and the cutoff conditions were padj < 0.05 and |log2FoldChange|> 0.58. The survival R package was used to perform univariate Cox regression analysis on differentially expressed genes, and the significant prognosis-related genes were identified with *p* < 0.05 as the threshold.

### Establishing a ferroptosis activation-related risk score model with machine learning

The cv.glmnet function of the lars package was used to perform least absolute shrinkage and selection operator (LASSO) analysis on the samples and the corresponding genes. The parameter used in LASSO analysis was *p* < 0.05. After screening, genes were included in the final model if the coefficients did not equal 0. FeAScore (ferroptosis activation-related risk score) = $$\sum_{\dot{I}=1}^{n}\begin{array}{c}expression of gene i\times lasso coefficient of gene i\\ \end{array}$$.

### Overall survival and prognosis prediction

Based on the median FeAS score, we divided the samples into high and low FeAS groups. The Kaplan‒Meier algorithm was used to predict the overall survival difference between the high and low FeAS groups. The receiver operating characteristic (ROC) curve and area under the curve (AUC) value were used to verify the accuracy and efficiency of the FeAS model.

### Prediction of biological functions

GO/KEGG enrichment analysis based on the gene set variation analysis (GSVA) algorithm was performed using bulk RNA sequence data. Classic GO/KEGG enrichment analysis was also performed using single-cell RNA sequencing data. Gene set enrichment analysis (GSEA) was performed using both datasets to reveal the difference between the high and low FeAS groups.

### Immune infiltration landscape

The ESTIMATE algorithm was used to describe the infiltration of immune cells and stromal cells. Analysis of tumor immune microenvironment cellular components was carried out using the previously reported ssGSEA algorithm, CIBERSORT algorithm, and xCELL algorithm. Timer 2.0 was used to analyzed the relationship between the selected genes and different immune cells (http://timer.cistrome.org).

### RNA pseudotime analysis and cell communication in tumor cells

The RNA pseudotime analysis in breast cancer cells was performed using monocle3 (PMID: 30,787,437). The different states of breast cancer cells were mapped to show their internal transformations. Communications between immune cells and breast cancer cells were analyzed using the R packages celltalker and Cellchat. Celltalker was used to identify different ligand‒receptor pairs.

### Establishing the regulatory network of transcription factors

The regulatory network of transcription factors was established using the RcisTarget database (https://resources.aertslab.org/cistarget/). The R package "SCENIC" was used to build the network. The AUCell algorithm was applied to evaluate transcription factor activation and identify regulon modules based on the connection specificity index.

### Correlation analysis of FeAS and drug sensitivity

We downloaded drug susceptibility data from cancer drug susceptibility genomics (GDSC) for approximately 1000 cancer cell lines (http://www.cancerrxgene.org). The half maximal inhibitory concentration (IC_50_) of antitumor drugs in cancer cell lines was used as the drug response index. We used Spearman correlation analysis to calculate the correlation between drug sensitivity and FeAS score using | Rs |> 0.2, *p* < 0.05. The same analysis was carried out in CCLE (https://depmap.org/portal/download/).

### GO and KEGG enrichment analysis

We used the clusterProfiler package to identify the GO and KEGG pathways in which the genes were enriched (pAdjustMethod = 'BH', pvalueCutoff = 0.05, and qvalueCutoff = 0.2).

### Statistical analysis

The Wilcoxon test was used for comparisons. The ROC curve verified the efficiency of the FeAS model. The samples were separated into two groups by the median FeAS score. The survival curve was plotted by the Kaplan‒Meier method to analyze the prognosis. The logarithmic rank test was used to determine the significance of differences. To assess whether the FeAS score is an independent predictor, we performed multivariate Cox regression model analysis with age, sex, and stage as variables. The statistical analysis was bilateral, with *p* < 0.05 indicating statistically significant differences (ns: *p* > 0.05, *p <  = 0.05, **p <  = 0.01, ***p <  = 0.001, ****p <  = 0.0001).

## Results

### Consensus clustering analysis of breast cancer samples and prognosis between clusters

**Fig. 1:**
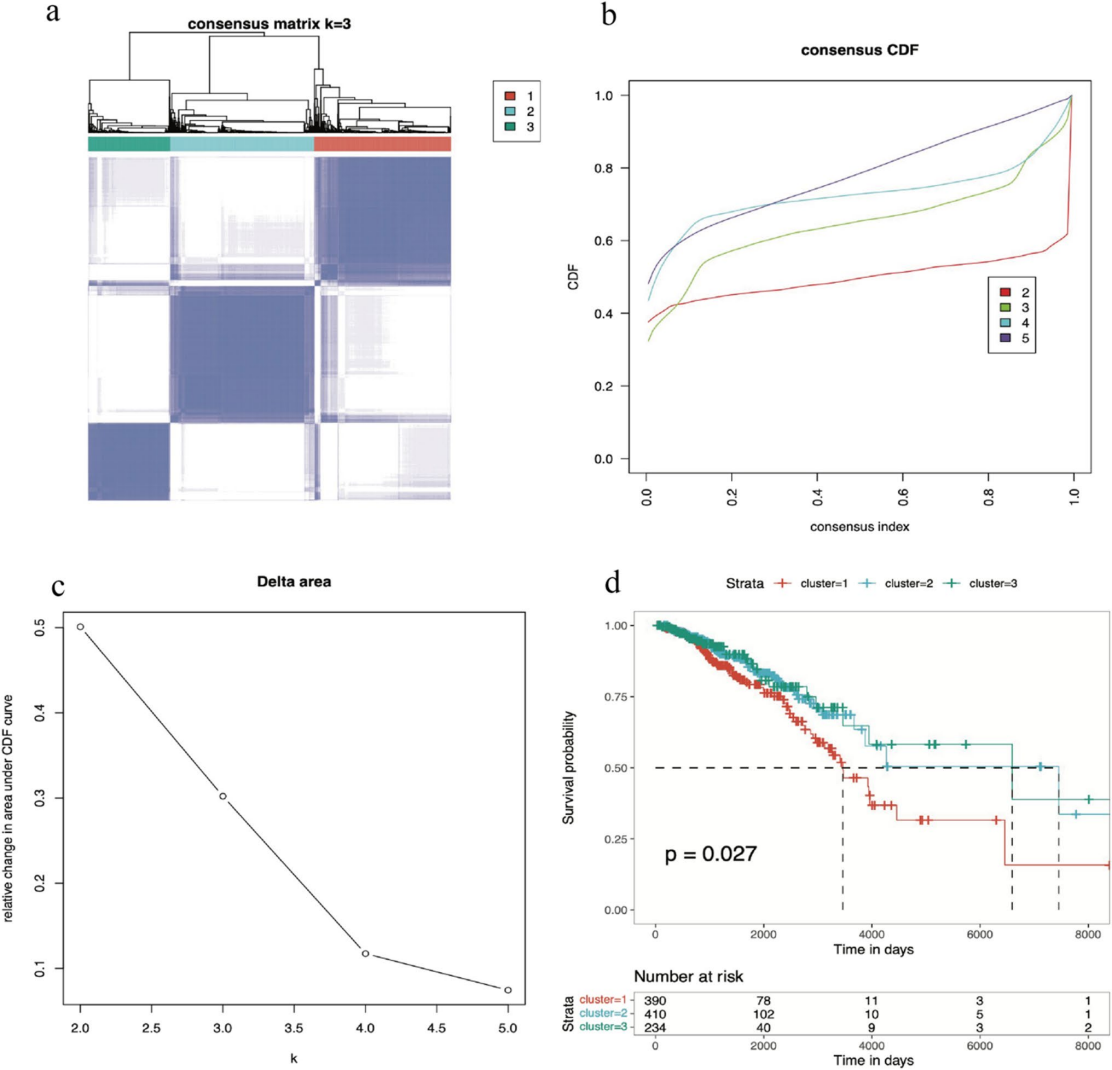
Consensus clustering analysis of the breast cancer samples and prognosis between clusters. **a** Cluster breast cancer patients in the TCGA-BRCA set according to the expression level of ferroptosis-related genes. **b** The CDF curve was used to determine the best method of clustering. **c** Data used to measure the quality of clustering results. **d **The prognosis among the three clusters

According to the consensus clustering analysis of ferroptosis-related genes, we divided the breast cancer samples within the TCGA dataset into three clusters, as shown by a matrix heatmap (Fig. 1a). The consensus cumulative distribution function (CDF) curves of the consensus matrix were generated to verify the cluster effect under different k values (Fig. 1b). When k was 3, the best cluster effect was observed. A delta area was used to show the relative change in the area under the CDF curve (Fig. 1c). Furthermore, based on the three newly defined clusters, Kaplan‒Meier analysis was used to show the survival probability among the three clusters, and the survival possibility of these clusters was significantly different (Fig. 1d).

### Combination of clusters and their characteristics

**Fig. 2:**
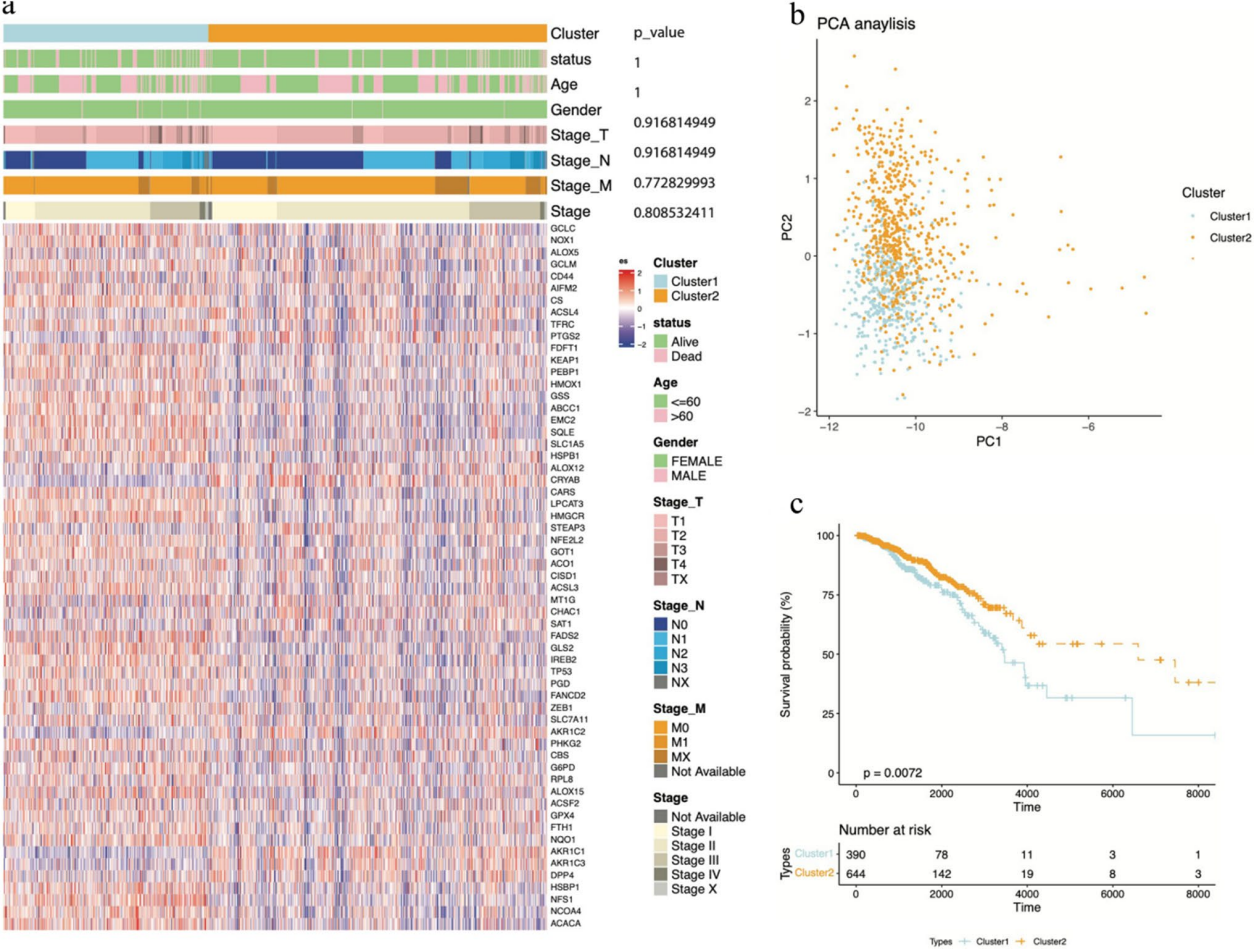
The expression of ferroptosis-related genes in the clusters and the prognostic features and clinical characteristics. **a** Heatmap of ferroptosis-related gene expression levels. **b** The result of PCA. **c** The prognostic difference between clusters

Based on the clustering results, we merged clusters 2 and 3 into a new cluster. The expression of ferroptosis-related genes in the two clusters is shown using a heatmap in addition to various clinical characteristics (Fig. 2a). Based on the heatmap, it was apparent that the expression of ferroptosis-related genes was significantly different between the two clusters, but no obvious differences in clinical characteristics were observed. The PCA revealed that the two clusters had a good degree of separation (Fig. 2b). Moreover, the Kaplan‒Meier analysis showed a distinct survival probability between them (Fig. 2c).

### Identification of differentially expressed genes

**Fig. 3:**
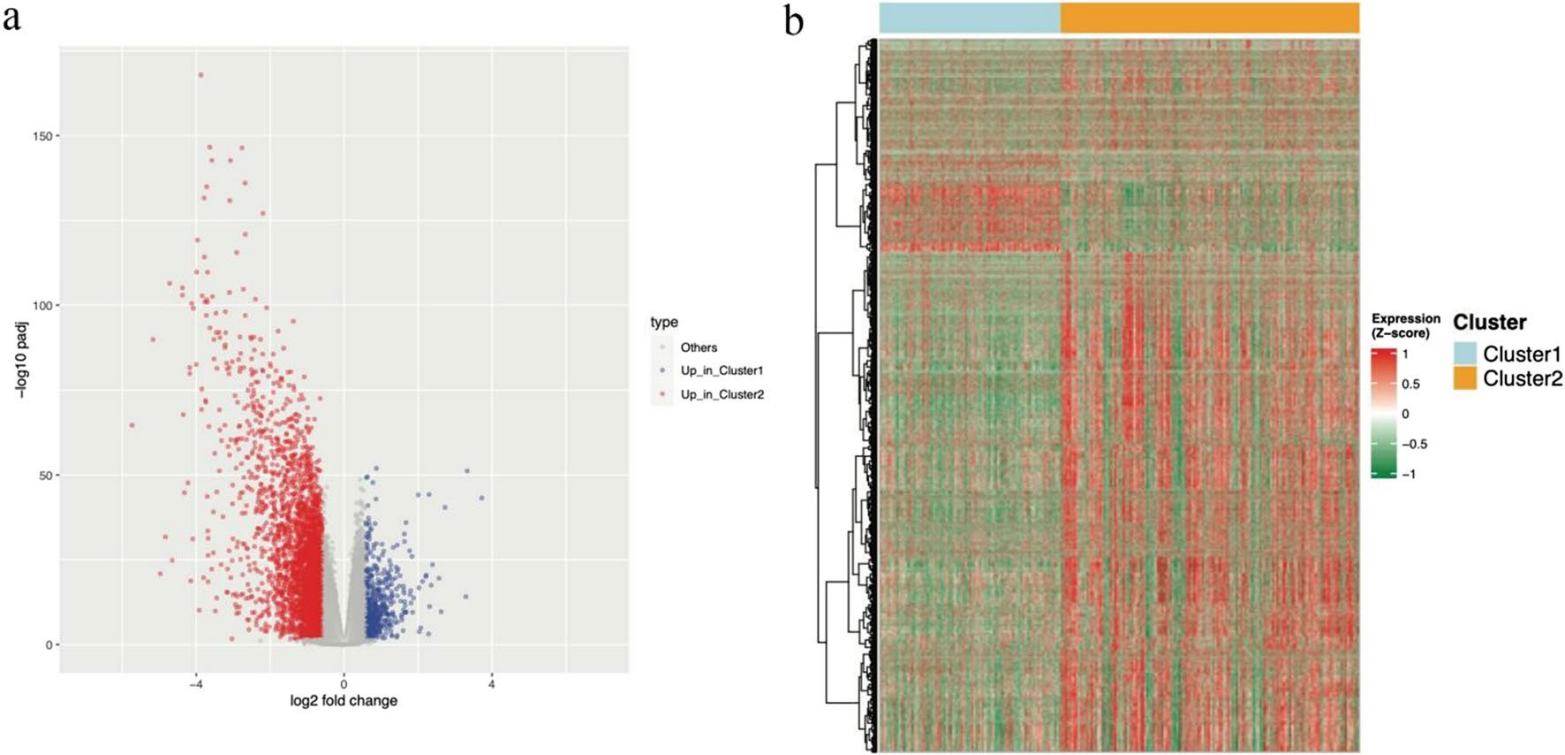
Analysis of differentially expressed genes. **a** Volcano plot of differentially expressed genes. **b** Heatmap of the levels of differentially expressed genes

We identified the differentially expressed genes between the two clusters divided based on the expression of ferroptosis-related genes (padj < 0.05, |log2FoldChange|> 0.58) (Fig. 3a). Specifically, 488 genes in cluster 1 had higher expression levels, and cluster 2 had 3094 genes with higher expression (Fig. 3b).

### Identification of prognosis-related genes

**Fig. 4:**
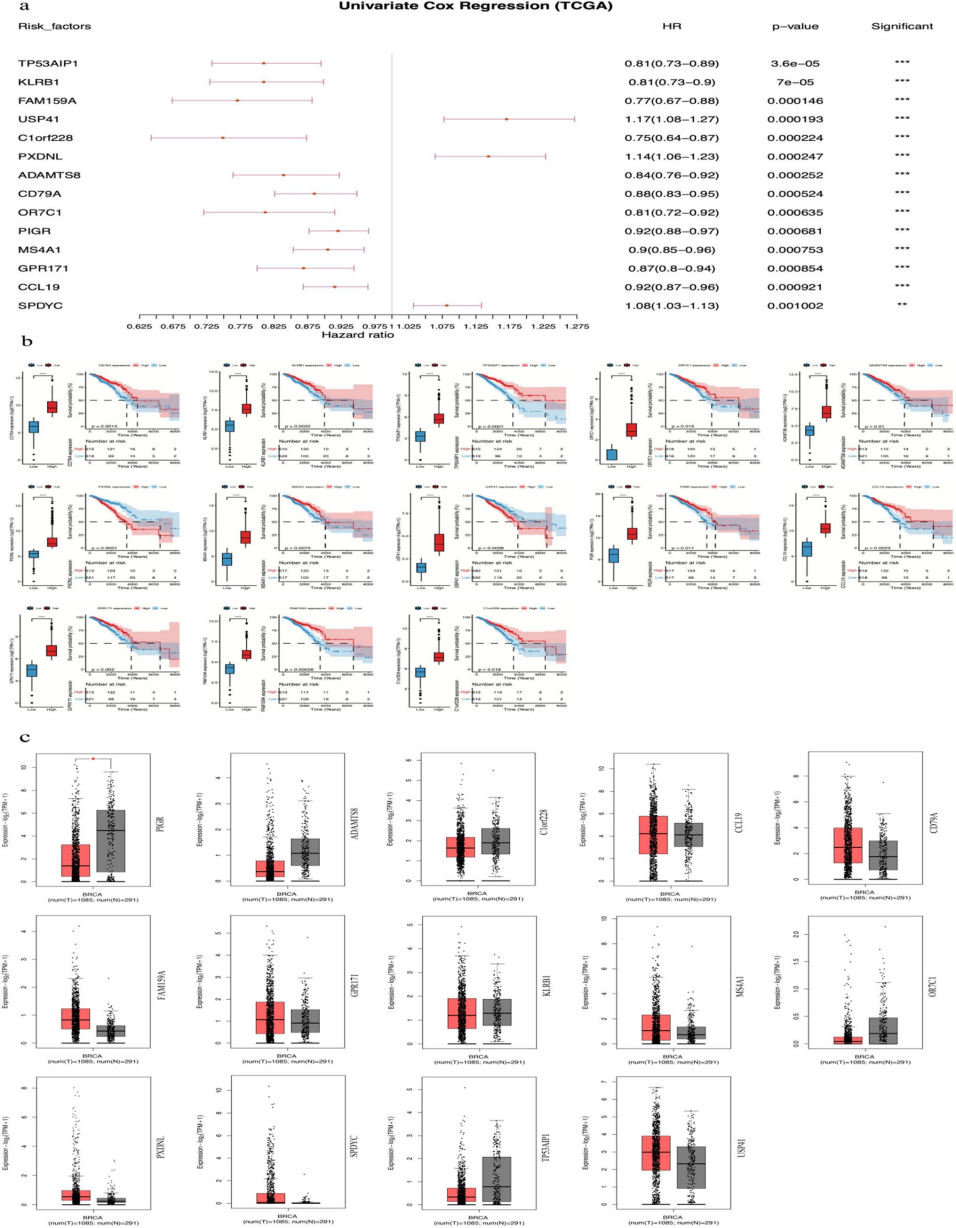
The results of the univariate Cox analysis of prognosis-related genes. **a** Forest plot of the univariate Cox analysis result. **b** Genes with significant prognostic differences between the high ferroptosis-activated group and the low ferroptosis-activated group. **c** Expression level of the 14 prognosis-related genes using bioinformatics analysis

According to the differentially expressed genes, univariate Cox regression analysis was used to reveal the correlation between these genes with prognosis. As a result, 14 genes were identified that had a significant correlation with prognosis. A forest plot was used to show the hazard ratio (HR) of these genes. Among them, the HR of USP41, PXDNL, and SPDYC was above 1, while the HR of the rest of the 14 genes was below 1 (Fig. 4a), which inferred that USP41, PXDNL, and SPDYC might be unfavorable prognostic factors, while others might be favorable. We also carried out bioinformatics analysis of these individual genes. USP41, PXDNL, and USP41 were charactered by higher expression trend in breast cancer tissue. Regarding the favorable factors, MS4A1 was found to be more highly expressed in normal tissue than in tumor tissue, and the rest of the genes were characterized by a higher expression trend in normal tissue but with no significant differences (Fig. 4c). By setting the median expression level as the baseline, we separated every gene into 2 groups, the high expression group and low expression group. Thirteen of them were associated with significant differences in prognosis between the high expression group and the low expression group, except SPDYC (Fig. 4b).

### Establishment of a ferroptosis-related risk model using machine learning analysis

The 14 screened prognosis-related genes were subjected to lasso-cox analysis. As a result, 13 of them were found to be significantly correlated to prognosis, while SPDYC was not. According to the results of the lasso-cox analysis, we established a ferroptosis activation-related risk score model (FeAS) that contained 13 genes as previously mentioned. We defined the product of the lasso coefficient of the gene and its expression as the ferroptosis activation risk score. The accumulated scores of the 13 genes defined the FeAScore.

### Good predictive ability of the FeAS model for prognosis

**Fig. 5:**
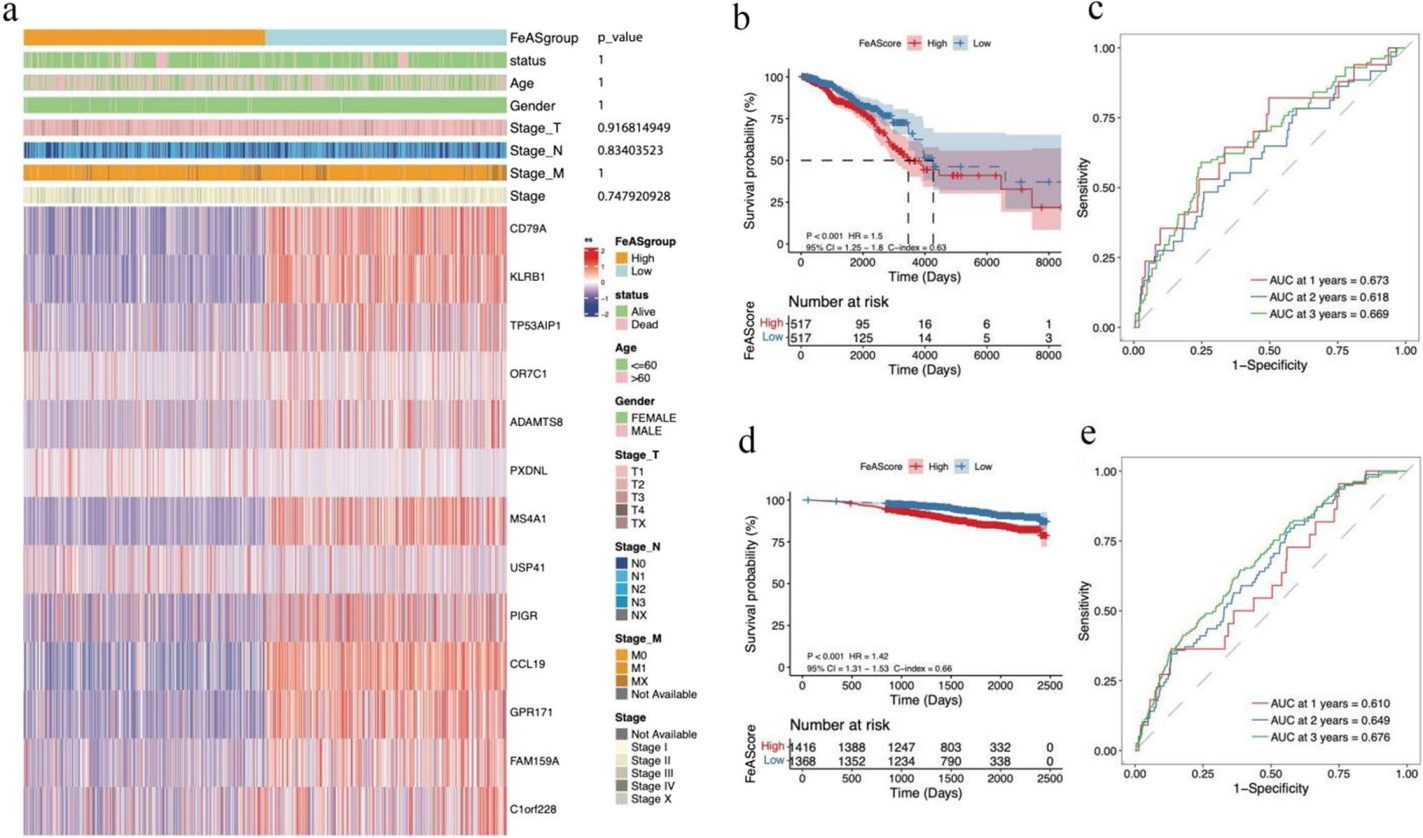
The prognostic difference between FeAS groups. **a** The relationship of the expression of selected genes and clinical features in a heatmap. **b** The differences in OS between FeAS groups in the TCGA-BRCA set. **c** The ROC curve of 1-year, 2-year, and 3-year survival in the TCGA-BRCA set. **d** The differences in OS between FeAS groups in GSE96058. **e** The ROC curve of 1-year, 2-year, and 3-year survival in GSE96058

According to the median FeAScore, we separated the samples from TCGA-BRCA into a high-risk group and a low-risk group. The expression levels of the 13 genes were significantly different between the two risk groups, as shown in a heatmap (Fig. 5a). However, for several clinical characteristics, there were no obvious differences between the two risk groups (Fig. 5a). Moreover, Kaplan‒Meier analysis was used to reveal the difference in the survival probability between the high score group and the low score group (Fig. 5b). The high score group was characterized by a poorer survival than the low score group. To further improve the FeAS model, we carried out ROC curve analysis to demonstrate the accuracy of predicting the 1-year, 2-year, and 3-year survival probabilities, and the AUC values were 0.673, 0.618, and 0.669, respectively (Fig. 5c), which proved that the accuracy of our risk model was good. Additionally, we verified our FeAS model in a validation dataset (GSE96058). The prognosis was significantly different between the high score group and the low score group in that the high score group had a poorer prognosis outcome than the low score group (Fig. 5d). Regarding the ROC curve, the AUC values at 1 year, 2 years, and 3 years were 0.610, 0.649, and 0.676, respectively (Fig. 5e).

### Further validation of the FeAS model

**Fig. 6:**
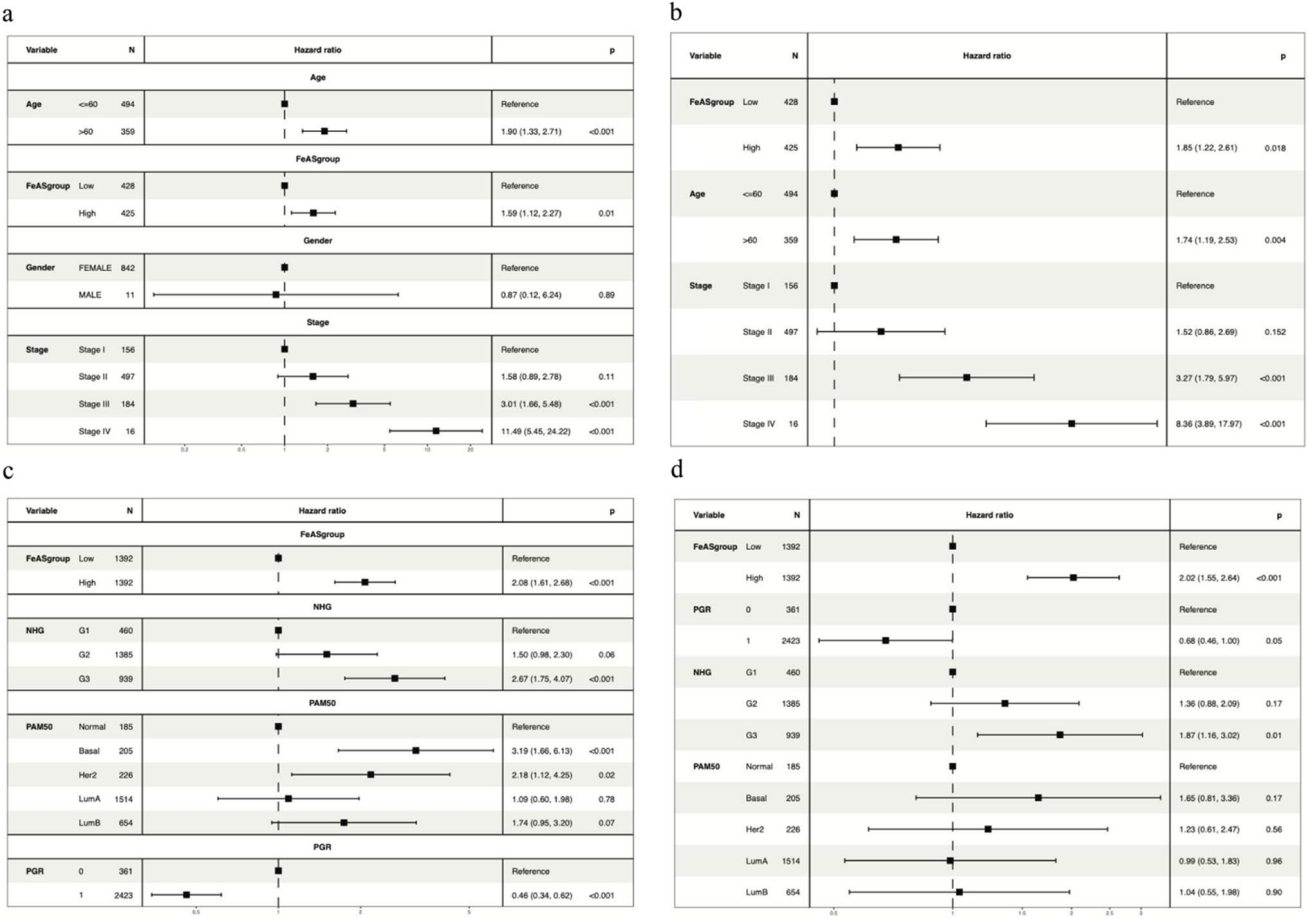
Univariate and multivariate COX analysis. **a** Univariate COX analysis in the TCGA-BRCA set. **b** Multivariate COX analysis in the TCGA-BRCA set. **c** Univariate COX analysis in GSE96058. **d** Multivariate COX analysis in GSE96058

Based on the univariate Cox regression analysis, the HR for a high FeAS score was 1.90, *p* < 0.001. Moreover, the HR was 1.85 for a high FeAS score in multivariate Cox regression analysis. In conclusion, FeAS could be regarded as an independent prognostic factor (Fig. 6a and b). In addition, we proved our conclusion in the GSE96058 validation set with univariate Cox and multivariate Cox regression analyses as the HRs were 2.08 (*p* < 0.001) and 2.02 (*p* < 0.001), respectively, and the FeAS model could be considered an independent prognostic factor (Fig. 6c and d).

### Analysis of FeAS model using single-cell RNA sequencing

**Fig. 7:**
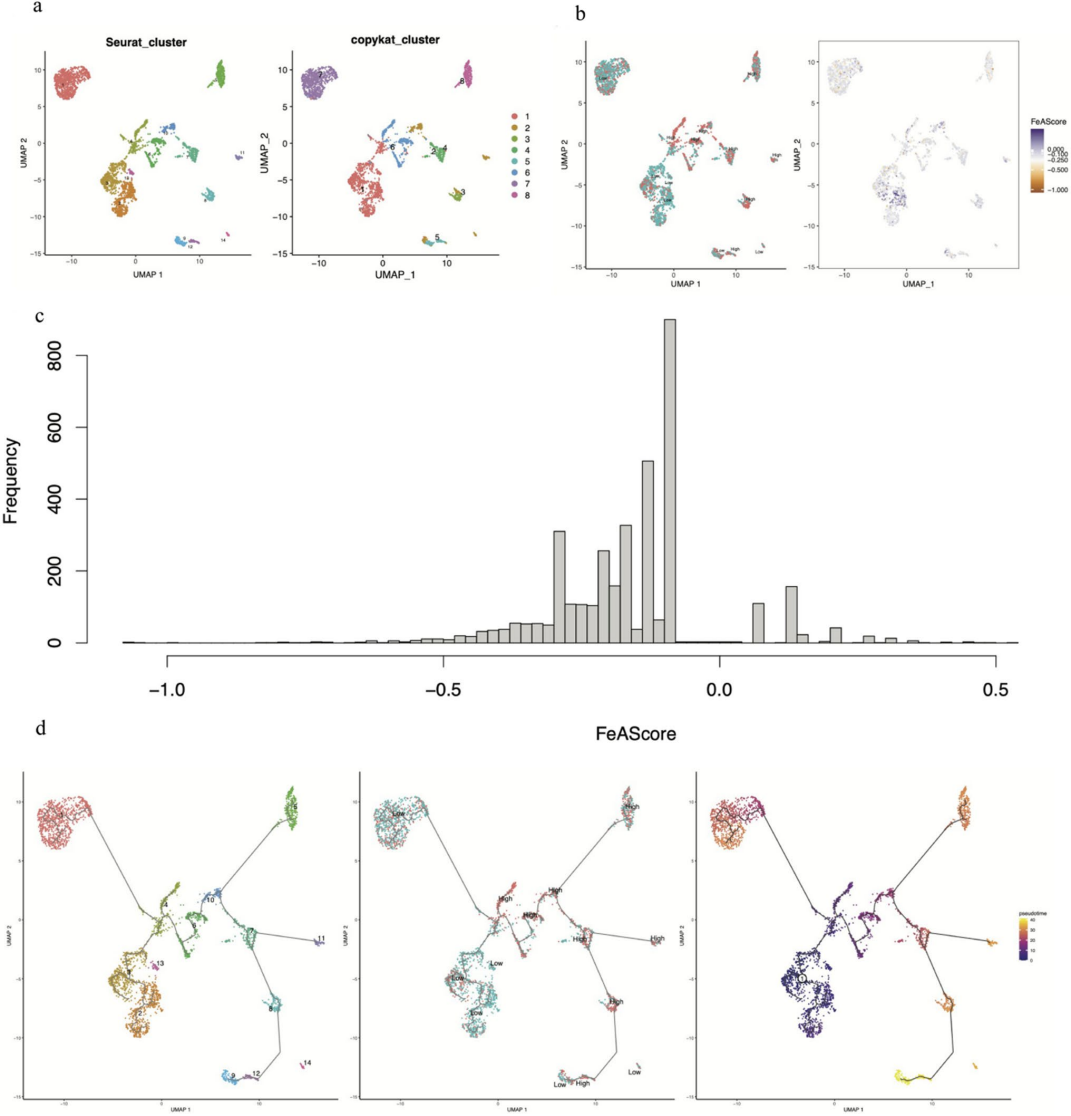
Single-cell clustering analysis and pseudotime analysis in FeAS groups. **a** Cluster subtypes of breast cancer cells and subtypes based on CNV. **b** The distribution of FeAS groups in breast cancer cells. **c** The frequency distribution of the FeAS score in breast cancer cells. **d** The pseudotime trajectory in breast cancer cells

Due to the limited amount of single-cell RNA sequencing data, the actual gene expression levels detected in each single cell varied, so we only used cells in which at least one gene from the 14 genes could be detected. As a result, 3710 of 20,473 malignant cells were identified. Thirteen subtypes of tumor cells were identified, and 8 subtypes were identified based on CNVs (Fig. 7a). For the 3710 cells, FeAS was used for calculations using the single-cell samples. Additionally, the median score was considered the baseline to define the 13 clusters as the high score group or low score groups (Fig. 7b). The distribution of the FeAS score was evaluated and mainly occurred between − 0.5 and 0 (Fig. 7c).

Moreover, according to the clusters defined by CNV, all 20,473 cells could be divided into 8 subtypes. Cluster 1 had less CNV than the remaining 8 clusters, which showed that these cells had a more normal status and were more likely to be normal cells. According to the sequential characteristics of genomic changes during the formation of cancers, we aimed to use cluster 1 to represent the beginning of the progression of carcinogenesis (Fig. S1).

As the pseudotime analysis performed using Monocle 3 showed, 13 clusters were identified by a pseudotime trajectory tree. Furthermore, the FeAS score was indicated as low or high in these clusters. The tumor clusters were also colored according to the analyzed pseudotime value (Fig. 7d). In conclusion, the increase in the FeAS score from low to high was in accordance with the progression of cancer.

### Differentially activated transcription factors between the high FeAS and low FeAS groups

**Fig. 8:**
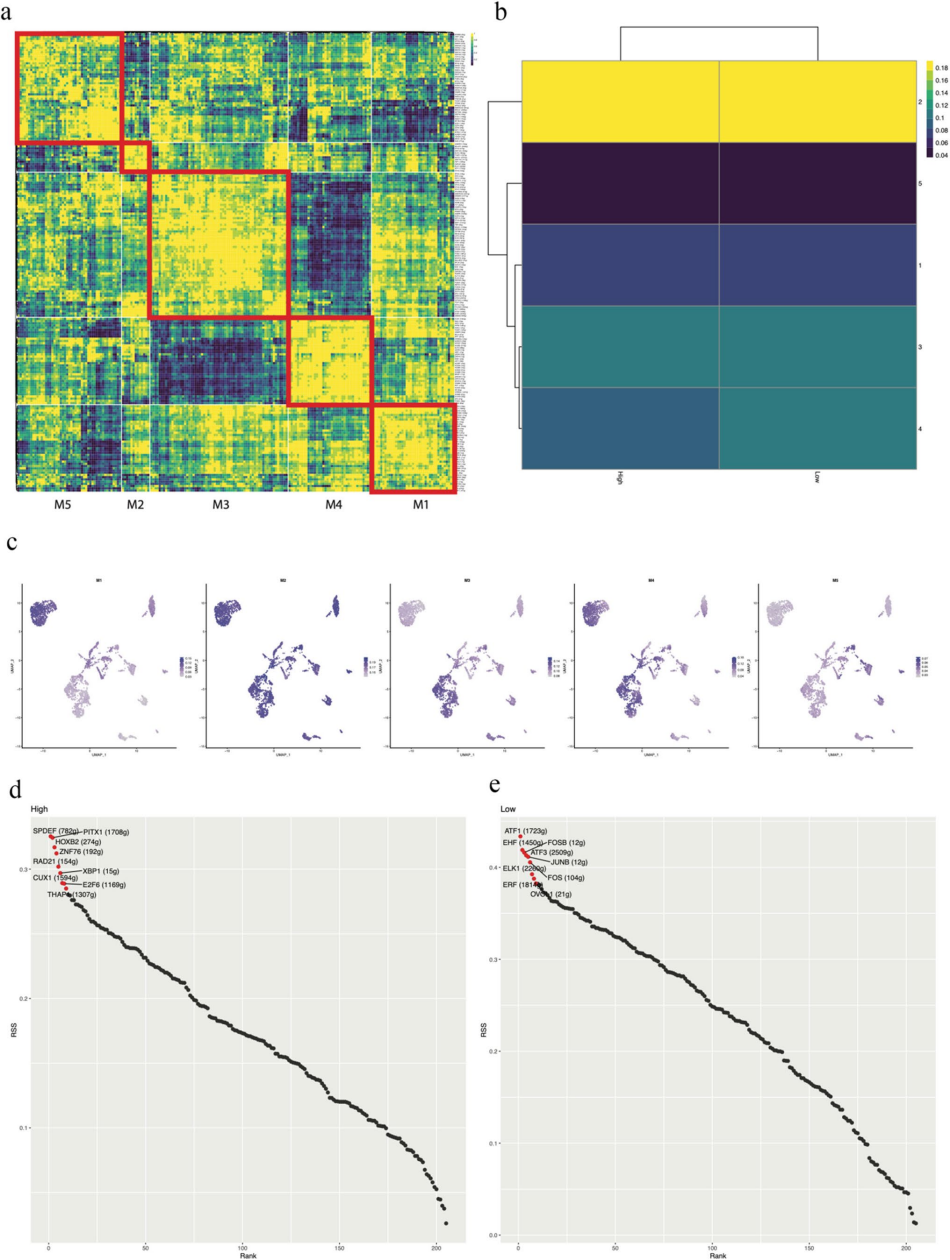
The differentially expressed transcription factors between the high and low FeAS groups. **a** According to the synergy of different transcription factors, breast cancer samples were divided into 5 modules. **b** The potential relationship between the FeAS scoring system and the module. **c** Distribution of AUCs of different modules in breast cancer cells. **d** and **e** The first 10 differentially activated TFs in the high and low FeAS groups

Furthermore, we explored the activation of transcription factors in breast cancer cells in two FeAS groups using a heatmap (Fig. 8a). As the regulation of gene expression is influenced by the interactions among various transcription factors, we analyzed 5 different clusters, M1-M5, in which every cluster represented a group of transcription factors that interacted with each other. Based on the connection specificity index, we calculated the regulon activity scores (RAS) of each cluster according to previous work (PMID: 30,404,000). We further analyzed the relationship between the RAS of 5 clusters and the 2 FeAS groups (Fig. 8b). As a result, M2 and M3 had relatively higher RAS in both the high and low FeAS groups. Moreover, the RAS of M1 and M5 was low in both groups. Regarding M4, there was a significant difference in RAS distribution between the high and low FeAS groups (Fig. 8b). The scores of the activity of regulons in single cells of the 5 clusters were calculated with AUCell and then shown by t-SNE plots (Fig. 8c). M4 was more activated in the low FeAS group. We also analyzed the top 10 differentially activated transcription factors in the high and low FeAS groups individually according to the regulon specificity score (RSS) (Fig. 8d and e).

**Fig. 9:**
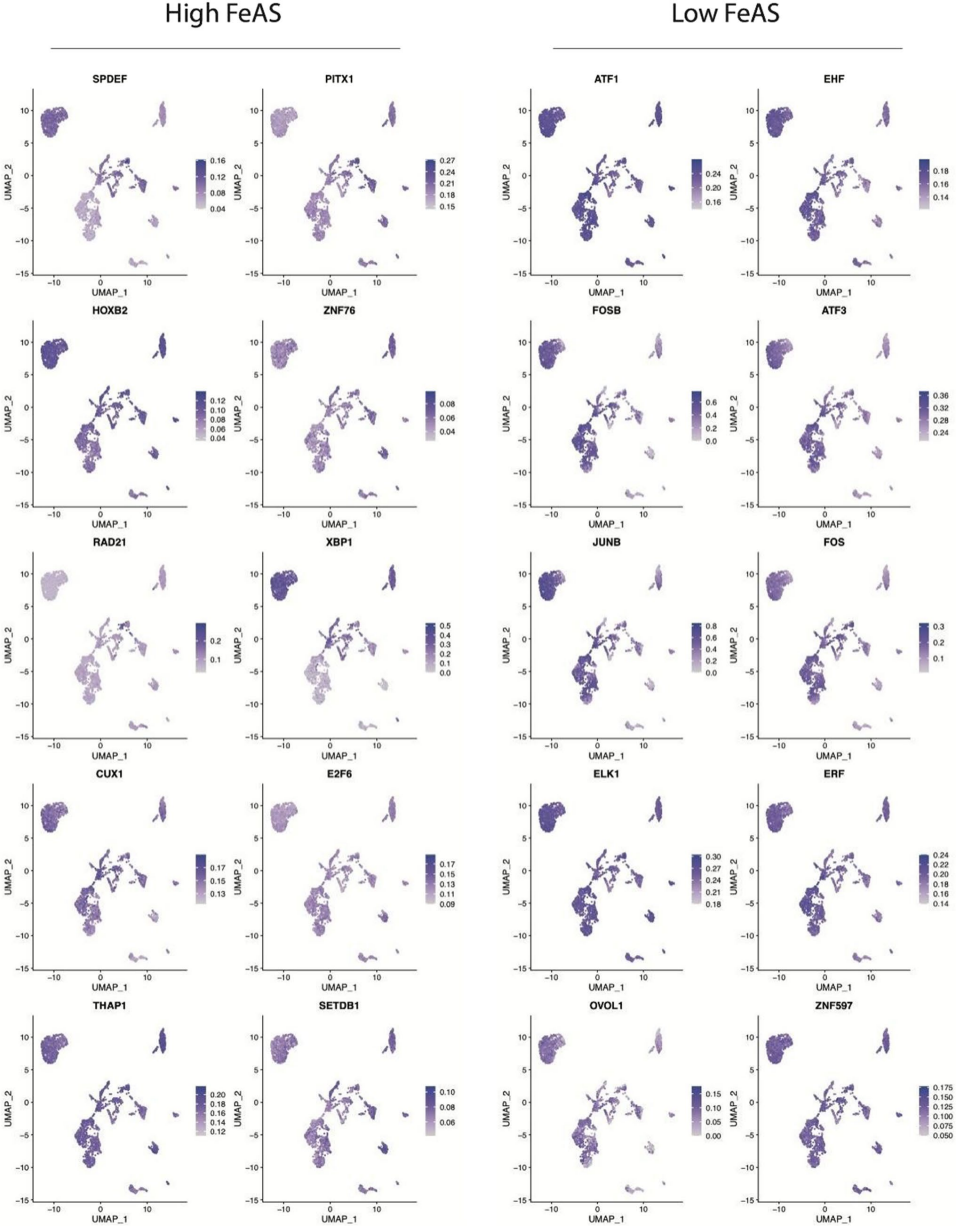
The enrichment distribution of the top 10 differentially activated TFs

The top 10 differentially activated transcription factors were selected individually from the high FeAS and low FeAS groups, and their enrichment distribution in the clusters of cancer cells was analyzed (Fig. 9).

**Fig. 10:**
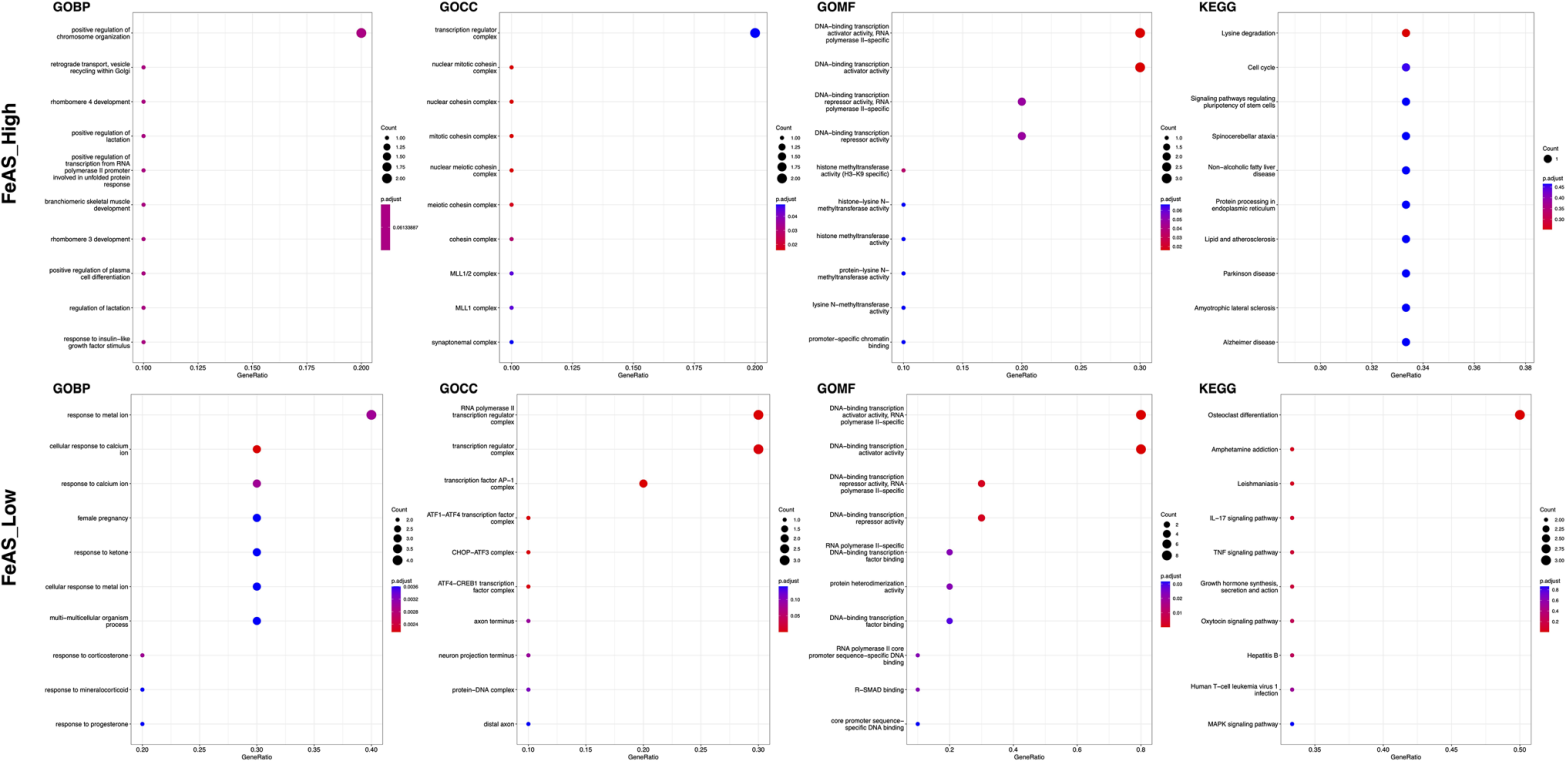
GO and KEGG enrichment analysis of the targeted genes of the top 10 differentially activated TFs

The transcription factor-corresponding regulons were used for GO enrichment and KEGG enrichment analysis. Regulons were selected according to a previous study in PMID32858223, and transcription factors that had fewer than 10 target genes were ignored. As a result, the top 10 activated transcription factors in the high FeAS group were significantly enriched in lysine degradation, transcription regulator complex, RNA polymerase II, and so on. In the low FeAS group, the top 10 activated transcription factors were significantly enriched in response to metal ion, transcription regulator complex, RNA polymerase II, IL-17 signaling pathway, TNF signaling pathway, and so on (Fig. 10).

The comprehensive enrichment results are shown in Fig. S2.

### Relationship between the immune microenvironment and the FeAS model

**Fig. 11:**
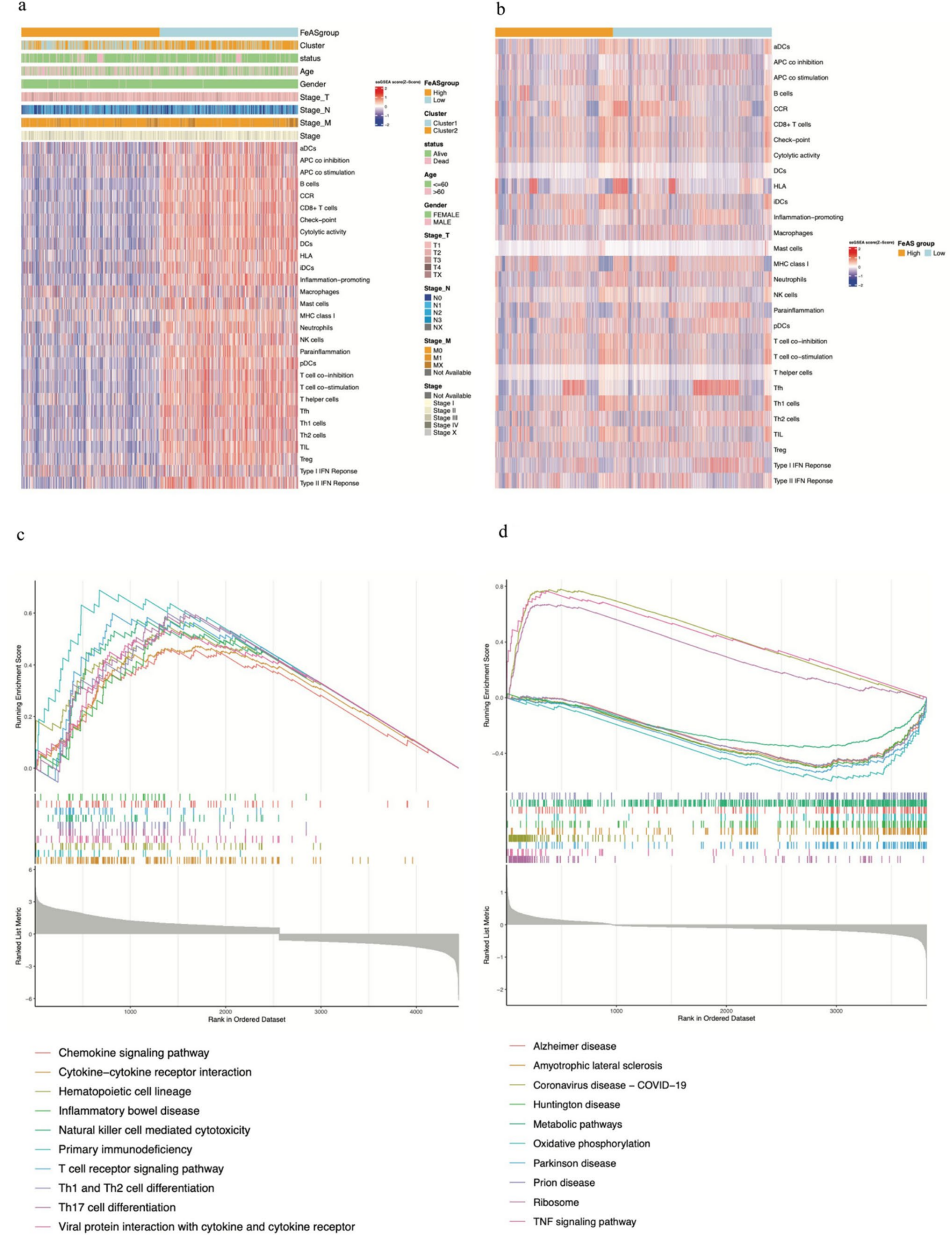
The enrichment of immune-related cells or pathways and GSEA using bulk and single-cell RNA sequencing data between the high and low FeAS groups. **a** Enrichment analysis of immune-related cells or pathways in the bulk data. **b** Enrichment analysis of immune-related cells or pathways in single-cell RNA sequencing data. **c** GSEA of the bulk data. **d** GSEA of single-cell RNA sequencing data

Based on the bulk data, we investigated the enrichment status of 29 immune-related pathways in the high FeAS and low FeAS groups using GSVA (10.1016/j.omtn.2021.06.024). As a result, the enrichment score of the 29 immune-related pathways in the high FeAS group was significantly lower than the score in the low FeAS group, which was clearly visualized in the heatmap (Fig. 11a). Using the same analysis method, we revealed the differences in the enrichment scores between the two FeAS groups using single-cell RNA sequencing and found that the score in the high FeAS group was also less than that in the low FeAS group (Fig. 11b). Both results were in high accordance with each other. Furthermore, we investigated the difference in GSEA between the two FeAS groups. Regarding the bulk data, the high FeAS group was significantly enriched in immune-related pathways (Fig. 11c), while the single-cell RNA sequencing data showed enrichment in the TNF signaling pathway, metabolism-related pathways, and so on (Fig. 11d).

**Fig. 12:**
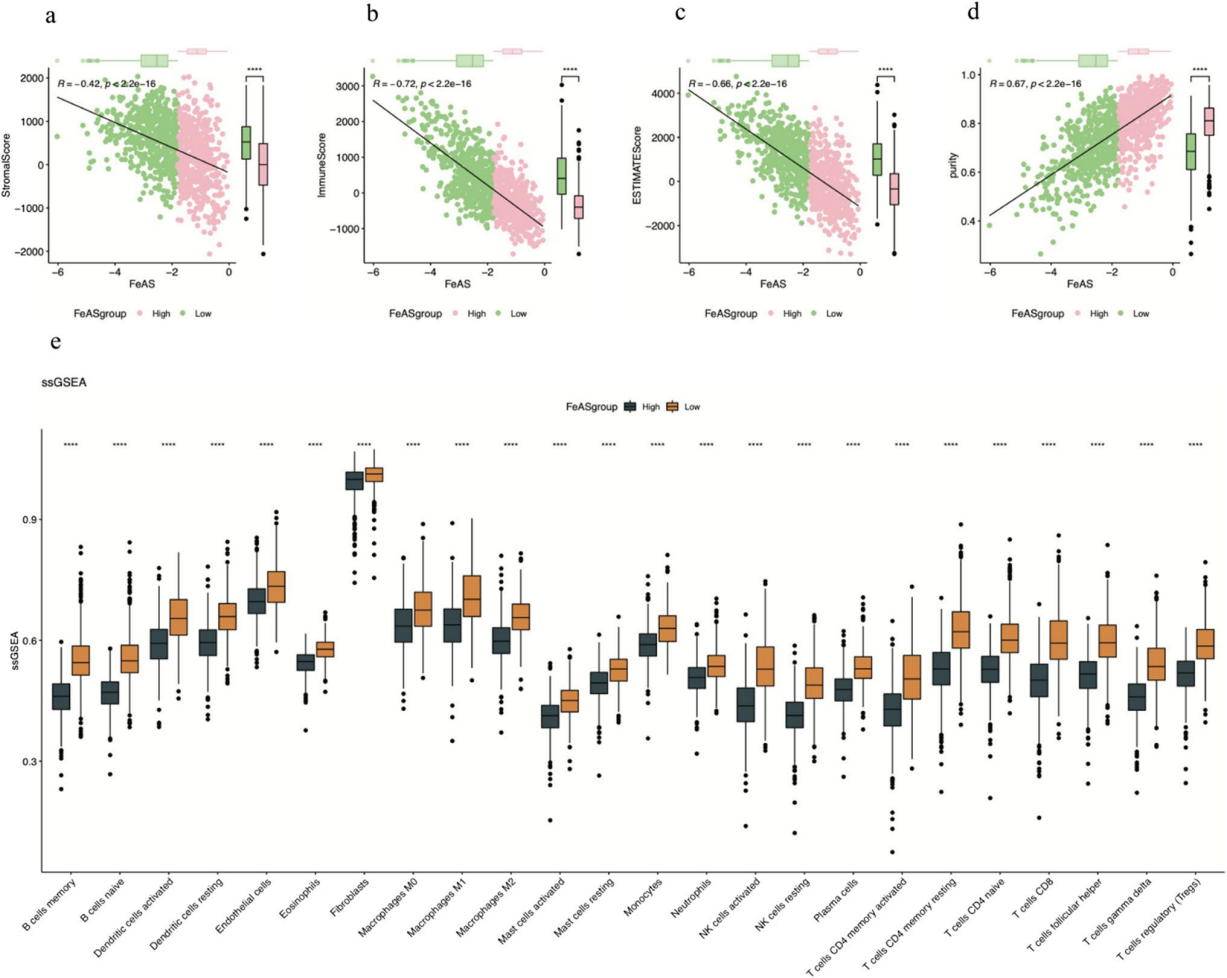
Immune infiltration landscape between the high and low FeAS groups. **a**–**d** The correlation between FeAS and stromal score, immune score, ESTIMATE score, and purity. **e** Analysis of immune cell infiltration based on ssGSEA

Furthermore, we revealed the differences in the landscape of immune infiltration between the high FeAS and low FeAS groups with ssGSEA. Surprisingly, the amounts of all kinds of immune cells in the high FeAS group were significantly lower than those in the low FeAS group (Fig. 12e–g). To further verify the result, we then used xCELL and Cibersort to perform the same analysis in TIMER2.0 (PMID: 32,442,275), which showed that the results were in accordance with the former finding (Fig. S3). Using TIMER2.0, we also analyzed the relationship between the selected genes and different immune cells (Fig. S4). Additionally, using ESTIMATE, the relationships between the FeAS score and StromaScore, ImmuneScore, ESTIMATEScore, and purity were individually revealed (Fig. 12a–d). The first three were negatively correlated with the FeAS score (*p* < 0.05), and purity was positively correlated with the FeAS score (*p* < 0.05).

### The relationship between special cell communication and the FeAS model

**Fig. 13:**
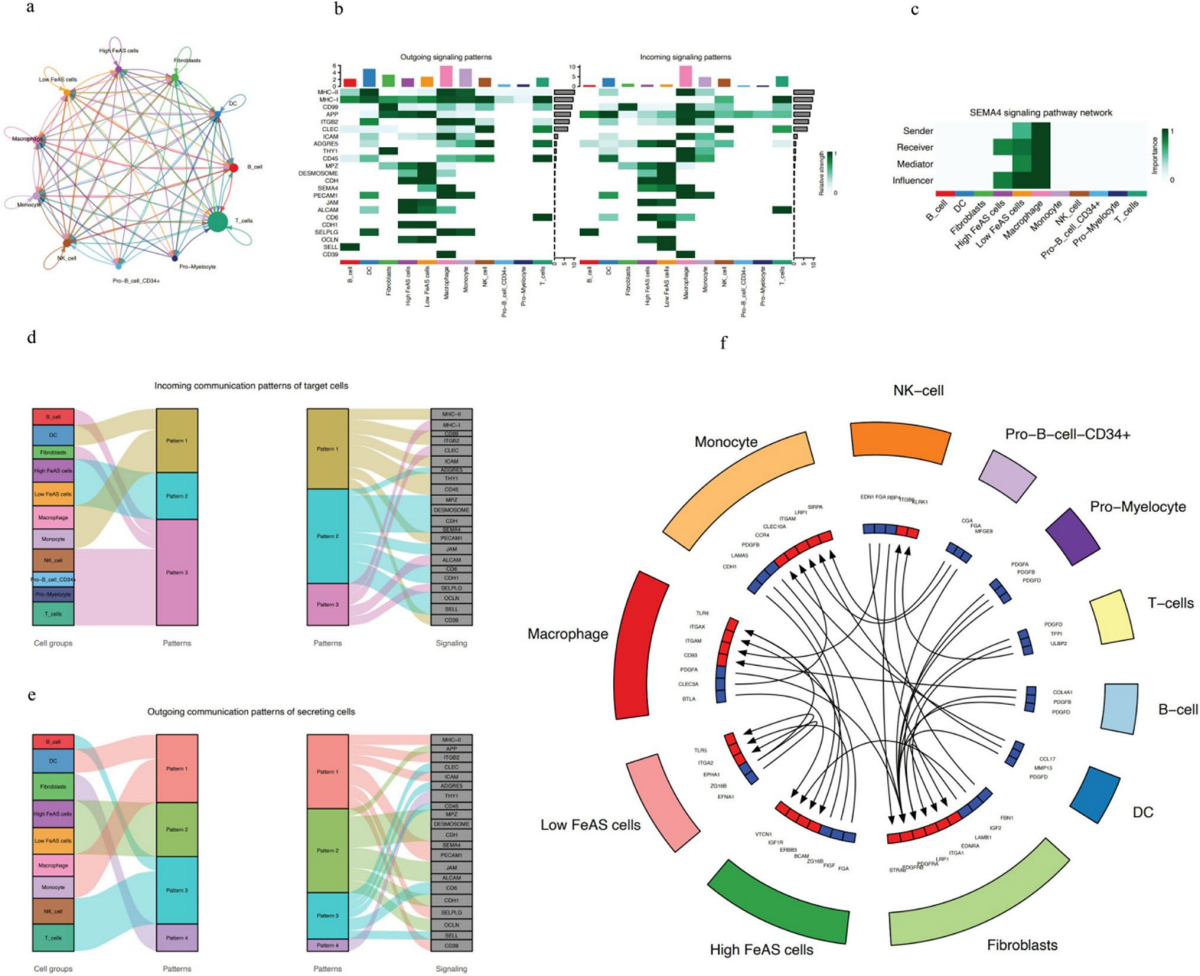
Cell communication between cells in the high and low FeAS groups and different immune cells or signaling pathways. **a**–**e** The complex cell communication among high or low FeAS breast cancer cells and different immune cells. **b** The correlation between FeAS groups or immune cells and different signaling pathways. **c** SEMA4 signaling pathway network. **d** Incoming communication patterns of target cells. **f** Receptor ligands among different cells

Based on the single-cell RNA sequencing data using CellChat, we analyzed the communication pattern between cancer cells and immune cells (Fig. 13a). As a result, there was complex communication among high or low FeAS cancer cells and immune cells. We found that there were specific communication patterns between high or low FeAS cells and immune cells, both in incoming and outgoing signaling patterns. As a result, the SEMA4 pathway had significantly different relative communication strength in the outgoing signaling pattern. However, in the incoming signaling pattern, the SELL and CDH1 pathways had significantly different relative communication strengths (Fig. 13b). Regarding the SEMA4 pathway, Sender, Mediator, and Influencer were characterized by significant differences between the high and low FeAS groups (Fig. 13c). Communication patterns between tumor cells and immune cells and signaling pathways corresponding to different patterns were shown in our further analysis (Fig. 13d and e). Furthermore, we used CellTalker to identify ligand‒receptor pairs that play roles during the communication between immune cells and high or low FeAS cells. The ligand‒receptor pairs were significantly different between the high and low FeAS groups: cells from the low FeAS groups communicated with cells from the high FeAS groups and macrophages, while cells from the high FeAS groups communicated with fibroblasts, monocytes, and macrophages (Fig. 13f).

### Different treatments between the high FeAS and low FeAS groups

**Fig. 14:**
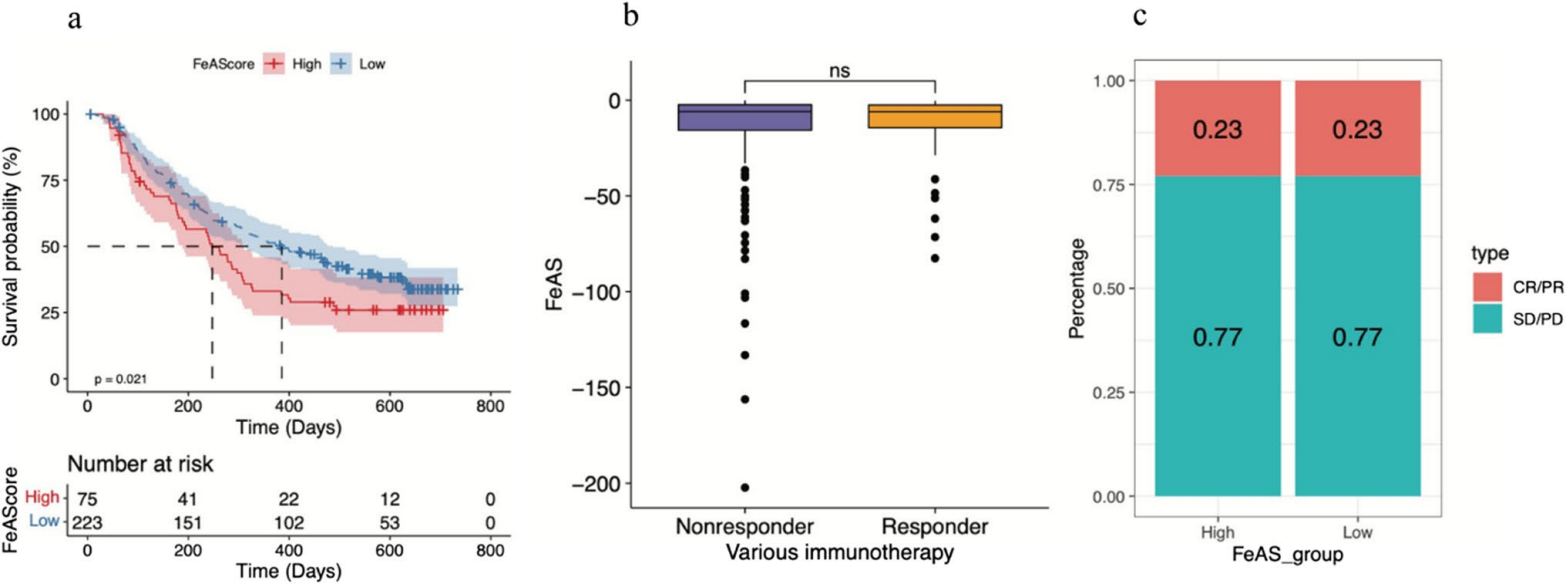
The treatment analysis between high and low FeAS groups based on IMvigor210. **a** Kaplan‒Meier analysis between FeAS groups. **b** The chemotherapy response between FeAS groups. **c** CR/PR and SD/PD between FeAS groups

First, based on the IMvigor210 dataset, we analyzed the ability of the FeAS model to predict chemotherapy efficacy. Setting the upper quartile as the baseline to divide the high and low FeAS groups, there was a significant difference in overall survival (OS) between the two groups (Fig. 14a). However, regarding the chemotherapy response, the FeAS value was not significantly different between the responder and nonresponder groups (Fig. 14b). Moreover, CR/PR and SD/PD in samples between the two FeAS groups were not significantly different (Fig. 14c).

In general, it could be concluded that the FeAS model had a good ability to predict overall survival after chemotherapy but not the chemotherapy response.

**Fig. 15:**
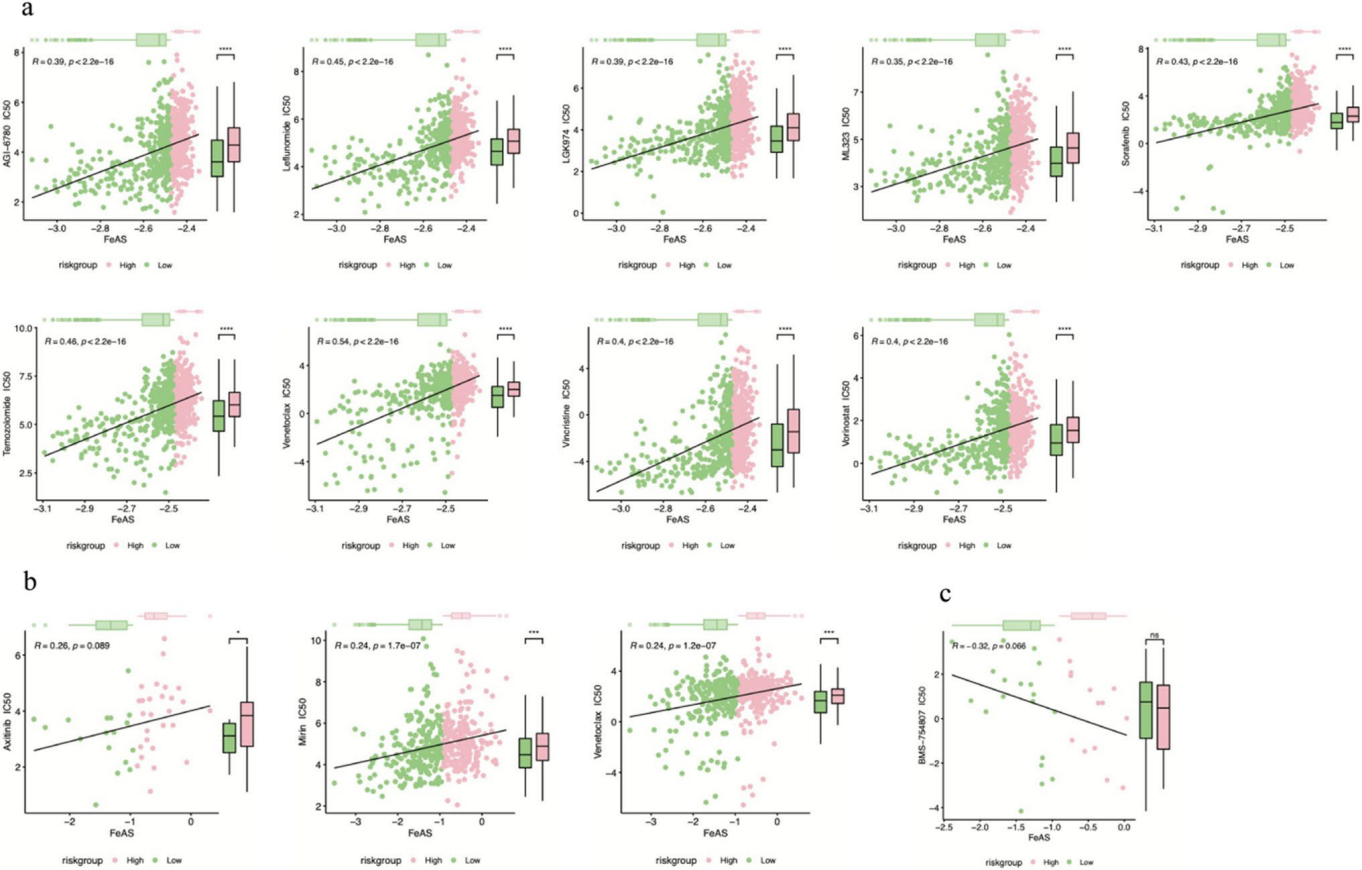
The correlation between FeAS and different drugs based on the GDSC and CCLE databases. **a** Drugs characterized by IC_50_ positively correlated with FeAS score in the GDSC database. **b** Drugs characterized by IC_50_ positively correlated with FeAS score in the CCLE database. **c** Drugs characterized by IC_50_ negatively correlated with FeAS score in the CCLE database

Furthermore, we revealed the differences in chemotherapy resistance between the two FeAS groups. First, we analyzed the IC_50_ of different drugs using the GDSC dataset and found that the IC_50_ values of 9 kinds of drugs were positively correlated with the FeAS value and had significant differences between groups (Fig. 15a). Drugs for samples in the high FeAS group were characterized by a higher IC_50_. Additionally, the same analysis was performed using the CCLE dataset, in which the IC_50_ values of 3 kinds of drugs were positively correlated with the FeAS value, and the IC_50_ value of 1 kind of drug was negatively correlated (Fig. 15b and c). In conclusion, drugs with IC_50_ values that were significantly correlated with FeAS had the same trend as that in high FeAS samples, and the drugs had higher IC_50_ values. This finding may infer that patients with high FeAS values exhibit stronger drug resistance.

## Discussion

As the most commonly diagnosed malignant cancer in women worldwide, breast cancer is a serious threat to human health (Wang et al. 2021). As of 2019, breast cancer accounted for 30% of newly diagnosed malignant cancers in women and accounted for 15% of deaths among women worldwide (DeSantis et al. 2019). Even with the development of better examination skills and the exploration of the molecular bases in breast cancer, complicated molecular mechanisms are involved in tumorigenesis and progression, and breast cancer is a distinctive disease with features of heterogeneous cell populations leading to treatment obstacles (Raman et al. 2016). Normal cells are characterized by limited survival times that follow the regular rule of aging and undergo cell death. Normal health relies on the ability to eliminate damaged cells, infected cells, and mutated cells, which is called regulated cell death (RCD) (Sauler et al. 2019). The increased exploration of RCD started when the term “apoptosis” emerged, but in recent years, many novel forms of nonapoptosis-regulated cell death have been identified, such as ferroptosis (Tang et al. 2019). Ferroptosis, as a special type of regulated cell death, is driven by iron-dependent phospholipid peroxidation, which is influenced by various kinds of cellular factors, such as redox homeostasis, iron handling, and mitochondrial activity (Jiang et al. 2021). It is also characterized by increased levels of intracellular reactive oxygen species (ROS) (Zhang et al. 2022). Moreover, ferroptosis has been confirmed to be related to tumor suppression, tumor immunity, and the vulnerabilities of cancer cells (Lei et al. 2022). Recent studies have revealed that ferroptosis is highly correlated with cancer treatment and drug resistance and that regulating ferroptosis will influence the effectiveness of therapy and even improve many conditions of drug resistance (Angeli et al. 2019; Li et al. 2020; Wu et al. 2020). Therefore, further investigation of how ferroptosis influences cancer heterogeneity and the microenvironment in breast cancer is warranted.

Single-cell RNA sequencing, as a novel cell analysis technology, has demonstrated its strong ability to explore cancer, revealing aspects of intratumor heterogeneity, the tumor microenvironment, metastasis, and therapeutic resistance (Lim et al. 2020). Previous studies have investigated ferroptosis in breast cancer, but most of them focused on the classic RNA-sequencing data linked to the TCGA-BRCA set to establish a model and reveal the roles of ferroptosis in breast cancer. We further relied on single-cell RNA sequencing to explore and analyze the potential mechanisms by which ferroptosis influences the microenvironment in breast cancer.

In our review, we systemically established an evaluation method called the ferroptosis activation score (FeAS). Based on the consensus clustering analysis of ferroptosis-related genes, we first divided the samples of the TCGA set into 3 clusters, and all these clusters were distinctive from each other. The survival probability of the three clusters was significantly different. To further improve the clustering, we merged clusters 2 and 3 into one single cluster as the new cluster 2. Using this approach, we completed the regrouping of the TCGA-BRCA set according to ferroptosis-related genes, which was helpful to focus the exploration of how ferroptosis influences breast cancer. Moreover, we analyzed the differentially expressed genes between the two clusters (padj < 0.05, |log2FoldChange|> 0.58). These identified genes were then further screened using univariate Cox analysis, and 14 genes were identified to be significantly associated with survival prognosis. Among them, USP41, PXDNL, and SPDYC were considered to be unfavorable prognostic factors. Recent studies have identified that USP41 is a potential factor that leads to the progression and migration of breast cancer cells (Huang et al. 2021). Other study revealed that USP41 knockdown inhibited cell proliferation, cell migration, and increased cell apoptosis in lung cancer (Ji et al. 2021). PXDNL, as a factor that is negatively correlated with breast cancer, has been rarely explored. It functions in basement membrane synthesis by promoting the formation of collagen IV crosslinks, but its other functions remain unknown (Kovacs et al. 2021). In other cancer phenotype, studies have showed that PXDNL knockdown inhibited EMT and motility of bladder carcinoma (Lu et al. 2023). SPDYC has been identified to be negatively correlated with the outcome of breast cancer and belongs to the speedy/Ringo cyclin-dependent kinase (CDK) family with functions in cell cycle transitions and progression (Cheng and Solomon 2008; Alsaleem et al. 2020). The rest of the 14 genes were favorable factors in breast cancer. Previous study showed that CCl19 suppressed angiogenesis through promoting miRNA-206 in colorectal cancer (Xu et al. 2018). All these findings provided evidence showing different roles of these selected genes in diffenet types of cancers. Exploration of miRNA alternation in cancer progression was also indeed that miRNA could influence the abnormal expression of different genes during cancer happening. After lasso-cox analysis, the FeAS evaluation model was established with the 13 screened genes using machine learning, and the model was then proven to be characterized by a good prognostic capability for breast cancer patients. We defined the FeAScore as ∑ of expression of gene × lasso coefficient of gene. According to the FeAS value, the median value was set as the baseline to divide the patients from the TCGA-BRCA set into a high-risk group and a low-risk group, and the genes included in the model had significantly different expression levels between groups. Patients in the high FeAS value group had a poorer survival probability than patients in the low FeAS value group, as shown by the Kaplan‒Meier curve. Furthermore, our FeAS model was verified using an ROC curve, and the AUC values indicated that the model was dependable. We also proved the validity of the FeAS model in the validation set GSE96058. The Kaplan‒Meier analysis still showed significantly different survival probabilities between the high FeAS and low FeAS groups. The AUC values at 1 year, 2 years, and 3 years were all above 0.61, which proved the reliability of the FeAS model.

As previously shown, the FeAS model was proven to be reliable in predicting the outcome of breast cancer patients. Moreover, the FeAS model could be applied not only to predict the outcome but also to reveal the cellular relationship between ferroptosis and the progression of breast cancer. With the application of single-cell RNA sequencing, we clearly revealed the heterogeneity of breast cancer. Based on the 13 screened genes, we selected 3710 single cells with single-cell RNA-quenching data, from which we identified 13 subtypes of breast cancer cells using the Seurat package. Seurat, as a useful workflow for analyzing single-cell RNA sequencing data, provides methods for data analysis and visualization (Mangiola et al. 2021). UMAP was used to visualize the subtypes of breast cancer cells after different analyses of single-cell RNA sequencing. As a technique for analyzing high-dimensional data, it is capable of providing the fastest run times, highest reproducibility, and the most meaningful organization of cell clusters (Becht et al. 2018). After calculation of the FeAScore as previously mentioned, we obtained the FeAS value of every single cell and set the median value as the baseline to divide all cells into high and low FeAS value groups. Furthermore, the 13 subtypes of breast cancer were all shown with their FeAS groups using UMAP for visualization. To reveal the relationship between the progression of breast cancer cells and the FeAS value (high or low FeAS value), the copy number variation (CNV) profile of 20,473 breast cancer cells was used for clustering analysis, and all cells could be divided into 8 subtypes shown with UMAP for visualization as well. CNV was defined as duplications or deletions of DNA segments that are greater than 1 kb compared to a reference genome (Sebat et al. 2004). It plays a vital role in cancer occurrence and progression and is responsible for gene aberrations in the human body (Bian et al. 2021). A previous study has already determined that CNV is capable of leading to the activation of oncogenes or inactivation of tumor suppressor genes that advance tumor development (Nik-Zainal et al. 2016; Hoang et al. 2018). In our study, different amounts of CNV among the 8 subtypes were shown within a heatmap, which indicated that cluster 1 was characterized by less CNV than the rest of the subtypes, which means that cluster 1 had more similarity with normal cells, and to some extent, cluster 1 could be regarded as the original differentiation from normal cells to cancer cells. Summarizing the results of the two different clustering analyses, cluster 1 based on CNV mostly consisted of samples from the low FeAS group. The pseudotime trajectory analysis also demonstrated the time-dependent progression process for breast cancer cells among different clusters, which was determined to be an original path combined with three branches by Monocel3. Monocle3 was used as a tool to map the cells onto a low-dimensional space with transcription states (Cao et al. 2019) and then cluster cells with similarity, merging adjacent clusters into supergroups (Wolf et al. 2019). Interestingly, the original path started from a cluster composed of low FeAS samples, which was also the start during tumorigenesis according to the CNV analysis. According to the CNV clusters, cluster 1 was the beginning of the timeline, while clusters 2, 4, and 6 were at the middle that differentiation then occurred. Cluster 5 could be regarded as the end of the timeline. In conclusion, we revealed the relationship between breast cancer cell development and the FeAS value. In single cells, the differentiation of cells was characterized along with increasing FeAS value, which proved that our FeAS model had predictive ability for the progression process during tumorigenesis and that the differentiation of FeAS in single cells had a gradually increasing trend.

To further reveal the difference in the potential microenvironment as the FeAS value changed, we investigated the activation of transcription factors in breast cancer cells. When interacting with each other, transcription factors play vital roles in regulating gene expression across time and space (Francois et al. 2020). These proteins are responsible for finely regulating transcriptional regulation and influencing organism phenotypes (Spitz and Furlong 2012). According to a previous study (Suo et al. 2018), we divided different transcription factors into 5 clusters, M1-M5, in which each cluster represented transcription factors that interacted with each other. We found that transcription factors in M2 and M3 were more activated in both the high and low FeAS groups, while M1 and M5 were less activated. Interestingly, the activation of M4 was different between the high and low FeAS groups. The finding involving M4 indicated that transcription factors in M4 might underlie one of the potential mechanisms through which ferroptosis influences breast cancer cells since they were more activated in the low FeAS group, which inferred that with increasing FeAS value, the activation of these transcription factors was suppressed. The detailed basic mechanism of this phenomenon is worth further exploration. The top 10 activated transcription factors were identified according to their regulon specificity score (Wu et al.) (Cabili et al. 2011), and their functional characteristics were further explored. Based on a previous study (Zhang et al. 2020), we selected the corresponding target genes of the top 10 activated transcription factors and performed GO and KEGG enrichment analyses. As a result, the top 10 activated transcription factors in the high FeAS group were significantly enriched in lysine degradation, transcription regulator complex, RNA polymerase II, and so on. In the low FeAS group, the top 10 activated transcription factors were significantly enriched in response to metal ion, transcription regulator complex, RNA polymerase II, IL-17 signaling pathway, TNF signaling pathway, and so on. It was revealed that the disturbance of transcription regulation occurred in both FeAS groups. According to previous research, the dysregulation of transcription leads to various diseases, including cancers (Soutourina 2018). IL-17 has already been determined to be responsible for its proinflammatory role in autoimmune disease, and the imbalance of its function leads to cancers and immune disease (McGeachy et al. 2019). It has been revealed that the overexpression of IL-17 signature genes is found in various cancers, such as cervical cancer, esophageal cancer, gastric cancer, hepatocellular carcinoma, and colorectal cancer (Le Gouvello et al. 2008; Miyahara et al. 2008). IL-17B and its receptor have been identified to be related to the progression and development of breast cancer (Alinejad et al. 2017). The IL-17 family consists of different protein members, and the activation of IL-17A and IL-17F has been proven to induce IL-6 signaling, activation of the NF-кB inflammatory pathway, and the signaling pathway of the STAT3 transcription factor, which further leads to the activation of acute inflammation, antimicrobial and antifungal defense molecules and the development of different kinds of cancers (Alinejad et al. 2017). Based on the above results, we determined the various functions of the IL-17 signaling pathway, and it may play a potential role during the process through which ferroptosis influences breast cancer cells, which needs further exploration. The TNF signaling pathway plays a vital role in various processes, including cell differentiation, cell proliferation, and apoptosis, and is involved in different metabolic processes. Regarded as having anticancer activity, TNF mediates the inflammatory response and regulates immune function to induce apoptosis of cancer cells (Chen and Goeddel 2002). For instance, TNF-α has been identified as a proinflammatory cytokine in the tumor microenvironment that influences different stages of breast cancer progression and patient survival conditions (Cruceriu et al. 2020). When TNF-α was first discovered in 1975, it was regarded as an antitumor cytokine (Carswell et al. 1975) that was able to facilitate the vascular destruction of cancer cells and exert a synergistic effect with chemotherapy at cancer sites (Seynhaeve et al. 2007; Daniel and Wilson 2008). However, with the in-depth exploration of TNF-α, it has been revealed that TNF-α also contributes to inflammation and cancer growth (Stathopoulos et al. 2007; Zins et al. 2007; Egberts et al. 2008). In this way, TNF-α is thought to act as a double-edged sword in either promoting or inhibiting cancer (Balkwill 2009). Studies in breast cancer indicated that TNF-α was detected at a higher level in invasive cancer than in benign tissue, while malignant breast cancer tissue had higher TNF-α levels (Leek et al. 1998). Thus, ferroptosis, as a novel kind of apoptosis, might have a potential correlation with the TNF signaling pathway based on our results, and the reduced activation of ferroptosis leads to a favorable effect of TNF. In breast cancer patients with low ferroptosis activation, TNF might be used as an antitumor treatment, but its basic mechanism needs further exploration.

To further reveal the variation in the microenvironment with increasing FeAS values, we analyzed the enrichment of immune-related pathways between the high FeAS and low FeAS groups using GSVA (Zhuang et al. 2021), which represented the activities or infiltration level of immune cells and pathways. Based on the heatmap, the activation of immune cells or immune-related pathways was significantly lower in the high FeAS group, which might suggest that high activation of ferroptosis in breast cancer cells may inhibit the immune capacity, leading to progression of breast cancer cells and a worse prognosis outcome. Previous studies have demonstrated that ferroptosis, controlled by different metabolic pathways, might result in an immunosuppressive microenvironment that facilitates cancer growth and progression (Angeli et al. 2019). It is already known that dying cells usually trigger apoptosis and induce communication with the immune system, sending messages to find and deal with these dying cells (Elliott and Ravichandran 2016). Ferroptosis, which is similar to apoptosis, might also promote signals that lead immune factors to the location of ferroptosis-related cell death, as a recent study revealed that ferroptotic tumor cells are engulfed by macrophages, which is convincing evidence (Kloditz and Fadeel 2019). It has also been revealed that the induction of ferroptosis in tumor cells is related to increased expression of PTGS2 and the release of PGE2 (Yang et al. 2014). With the increasing occurrence of ferroptosis, there will be a shift from anticancer activity to immunosuppression, and in this way, the overactivation of ferroptosis leads to the inhibition of the immune system in patients with cancers, which is also in accordance with our findings that patients with high ferroptosis activation levels exhibited the suppression of immune-related pathways. Moreover, we obtained the same conclusion based on the single-cell sequencing data. In addition, GSEA revealed differences in pathway enrichment between the high and low FeAS groups. Samples from the high FeAS group showed more enrichment of natural killer cell-mediated cytotoxicity, primary immunodeficiency, chemokine signaling pathway, T-cell receptor signaling pathway, Th1 and Th2 cell differentiation, Th17 cell differentiation, and so on, which indicated that the activation of ferroptosis could be able to influence the immune functions in breast cancer patients from the TCGA-BRCA set. Based on the single-cell RNA sequencing data, the enrichment results showed that the TNF signaling pathway, ribosome, and metabolic pathways were more activated in high FeAS samples. The immune infiltration landscape was revealed in our analysis using ssGSEA, and the infiltration of different immune cells and factors was lower in the high FeAS group than in the low FeAS group, which was still consistent with our previous conclusion. Moreover, we analyzed the relationship between these selected genes and different immune cells using TIMER2.0. For the genes as favorable factors, they were charactered by a positive correlation with different immune cells, for example, CD8 + T cell while the unfavorable ones were not which were in accordance with our previous exploration. Additionally, we analyzed the relationship between FeAS groups and StromaScore, ImmuneScore, ESTIMATEScore, and purity. Stromal cells and immune cells are the main components of the normal composition of cancer tissue and act as influencing factors in the progression of cancer cells. It has been revealed that stromal cells play important roles in the proliferation and progression of cancer cells (Kalluri and Zeisberg 2006; Hanahan and Weinberg 2011) and even influence drug resistance (Straussman et al. 2012). Infiltration of immune cells was confirmed to be related to tumor growth and metastasis in many kinds of cancers (Zhang et al. 2003; Mlecnik et al. 2011; Fridman et al. 2012). Therefore, analyzing the proportion of tumor-related stromal cells and immune cells is helpful to explore the potential mechanisms in cancer development. As the results show, the stromal score, immune score, and ESTIMATE score were negatively correlated with the FeAS value, which means that the higher the FeAS value is, the lower the proportion of tumor-related normal cells. The tumor purity was positively correlated with the FeAS value, which inferred that breast cancer patients with a high tumor burden were significantly characterized by higher activation of ferroptosis.

CellChat is a tool that can be used to analyze complex intercellular communication using single-cell RNA sequencing data and is able to predict the main input and output signaling among cells using network and pattern recognition (Jin et al. 2021). In our current research, we used CellChat in single-cell RNA sequencing data to analyze the potential intercellular communication between high or low FeAS groups and different kinds of immune cells to further reveal the difference between high and low FeAS groups. We determined that there was a significant difference in SEMA4 signaling between the high and low FeAS groups, and it was also involved in incoming communication patterns in both the high and low FeAS groups. Semaphorins, as a large family of secreted, transmembrane, or glycosylphosphatidylinositol-linked proteins defined by a semaphorin domain, have been determined to play important roles in different biological processes, such as the immune system, cardiovascular systems, and even cancer (Kolodkin et al. 1993; Luo et al. 1993; Takamatsu and Kumanogoh 2012; Gu and Giraudo 2013). SEMA4, which belongs to the semaphorin family, contains different subtypes that have been found to be involved in cancer and dendritic cells. These findings in addition to our current findings suggest that SEMA4 may have potential roles in breast cancer progression, as well as the activation level of ferroptosis. We also revealed a more detailed immune microenvironment involving cellular communication between the high and low FeAS groups, which provided insight into the role of ferroptosis in breast cancer occurrence and progression.

Finally, we analyzed the capacity of predicting chemotherapy efficacy using the FeAS model. As the results reveal, our model has a good ability to predict the OS of patients who receive chemotherapy, but the difference in chemotherapy response between the high and low FeAS groups was not significant, which inferred that even with differences in the activation of ferroptosis, the response to chemotherapy might not be influenced. However, regarding the relationship between ferroptosis and drug resistance, our study revealed a clinically relevant finding. Drugs with IC_50_ values that were positively correlated with the FeAS value had one common feature: the IC_50_ was significantly higher in the high FeAS group than in the low FeAS group. Based on this finding, we concluded that the high FeAS group was characterized by higher drug resistance, which indicated that the overactivation of ferroptosis-related genes plays a potential role in the progression of drug resistance. Many studies have examined the relationship between drug resistance and ferroptosis in breast cancer. It has been found that breast cancer cells that were resistant to lapatinib were sensitive to ferroptosis (Ma et al. 2017). Another study indicated that the suppression of the ferroptosis-related gene SLC7A11 seems to be connected with many kinds of tumor suppressor mechanisms, which results in an increased sensitivity to ferroptosis (Zhang et al. 2018). Nevertheless, how our FeAS model can be used and instruct treatment in breast cancer patients will need further validation in real-world clinical data.

Our current study had limitations. First, even though our FeAS model has been verified in different datasets with abundant analysis, further proof using real-world data is needed. Moreover, the potential basic mechanisms that play important roles during the progression of breast cancer cells according to pseudotime trajectory analysis should be further explored.

## Conclusion

In our current study, we aimed to determine the relationship between ferroptosis-related genes and breast cancer, and we regrouped breast cancer samples into novel clusters for analysis. Based on the differentially expressed genes between clusters, we established a ferroptosis activation score model that was characterized by good prognostic ability. Moreover, by using single-cell RNA sequencing data using machine learning, we systematically revealed the different landscapes in many aspects between the high and low FeAS groups, which included the tumor microenvironment, cell communication, immune infiltration, and drug resistance. All the aspects showed significant differences that verified our FeAS model as meaningful.

## Supplementary Information

Below is the link to the electronic supplementary material.

Figs. S1, S2, and S3 (DOCX 1207 kb)

Fig. S4 (PNG 3113 kb)

High resolution image (TIF 3078 kb)

## Data Availability

We proclaim that all the data and materials in our current research will be completely available to the public for scientific purposes without any commercial purposes that can breach participant confidentiality.

## Code availability

The web tools are available which have been listed in the manuscript. The key operations of using web tools or code have been explained in the manuscript.

## Abbreviations

*BRCA*: Breast cancer
*GO*: Gene ontology
*KEGG*: Kyoto Encyclopedia of Genes and Genomes
*ESTIMATE*: Estimation of stromal and immune cells in malignant tumors using expression data
*TCGA*: The Cancer Genome Atlas
*ROC*: Receiver operating characteristic
*GEO*: Gene Expression Omnibus
*FeAS*: Ferroptosis activation score
*ROS*: Reactive oxygen species
*AUC*: Area under the curve
*OS*: Overall survival
*RCD*: Regulated cell death
*PDAC*: Pancreatic ductal adenocarcinoma
*LASSO*: Least absolute shrinkage and selection operator
*GSEA*: Gene set enrichment analysis
*GSVA*: Gene set variation analysis
*ssGSEA*: Single sample gene set enrichment analysis
*CDF*: Cumulative distribution functions
*PCA*: Principal component analysis
*CNV*: Copy number variations
*RAS*: Regulon activity scores
*t- SNE*: T-distributed stochastic neighbor embedding
*RSS*: Regulon specificity score
*CDK*: Cyclin-dependent kinase
