# Supplementary material for: Application of single-cell RNA sequencing analysis of novel breast cancer phenotypes based on the activation of ferroptosis-related genes
Source: Funct Integr Genomics. 2023 May 22;23(2):173. doi: 10.1007/s10142-023-01086-0 (PMC10203036; doi:10.1007/s10142-023-01086-0)
Supplement: Supplementary file 1 — (DOCX 1207 kb) [file 10142_2023_1086_MOESM1_ESM.docx]

Figure S1: Heatmap showed the different clusters according to the CNV.


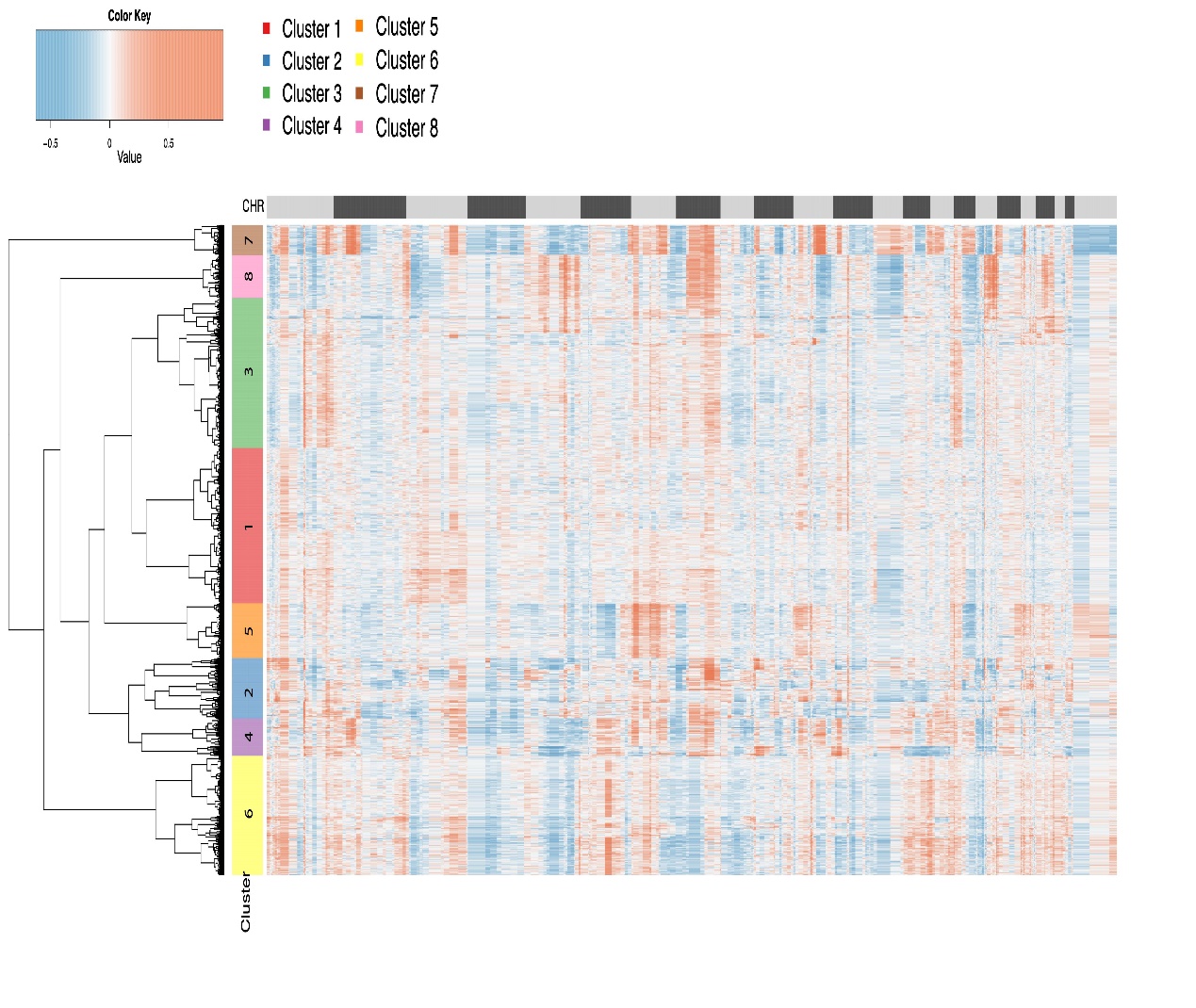


Figure S2: The GO and KEGG enrichment analysis based on the whole related genes.


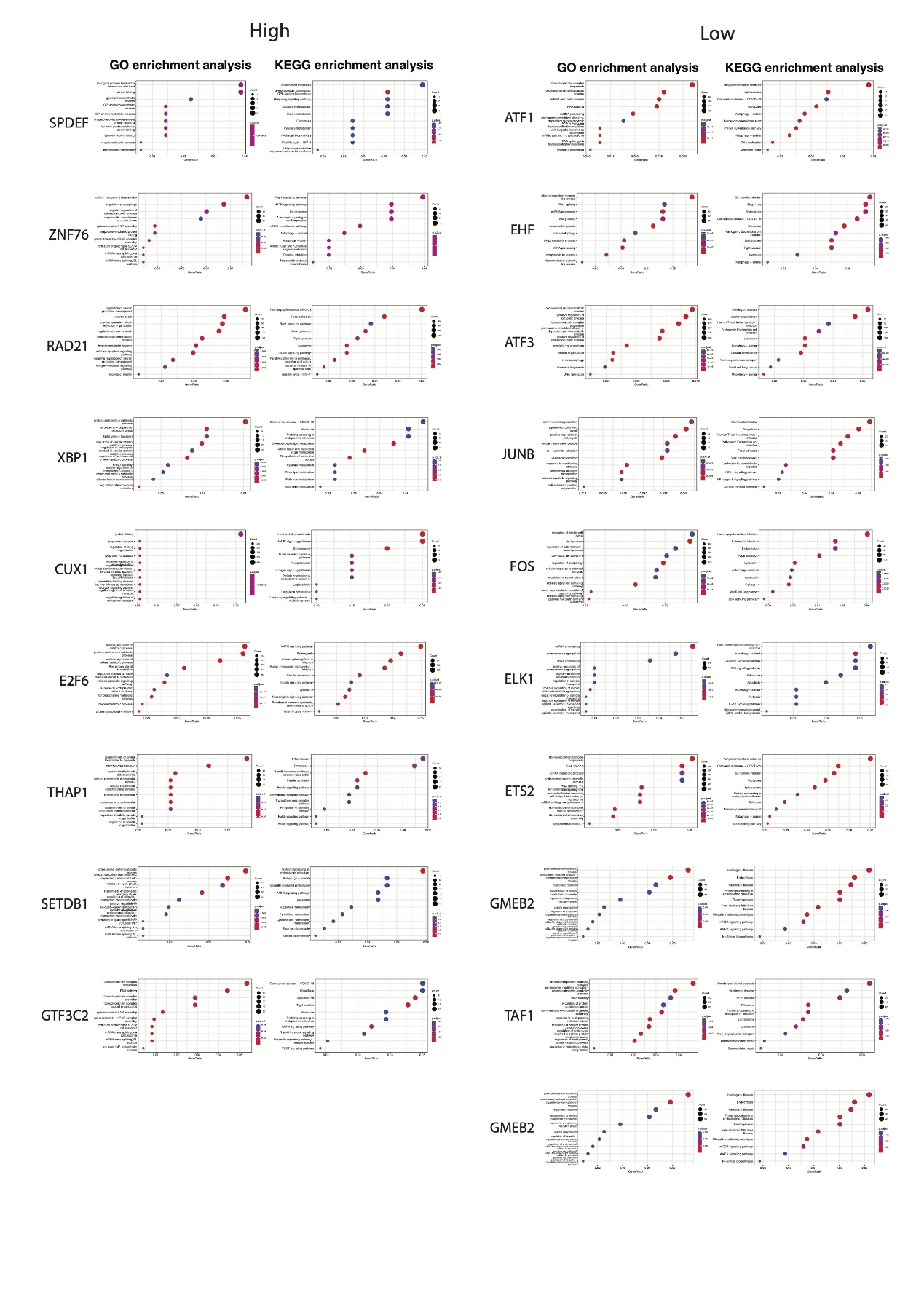


Figure S3: The different immune landscape between high and low FeAS groups using XCELL and CIBERSORT.**
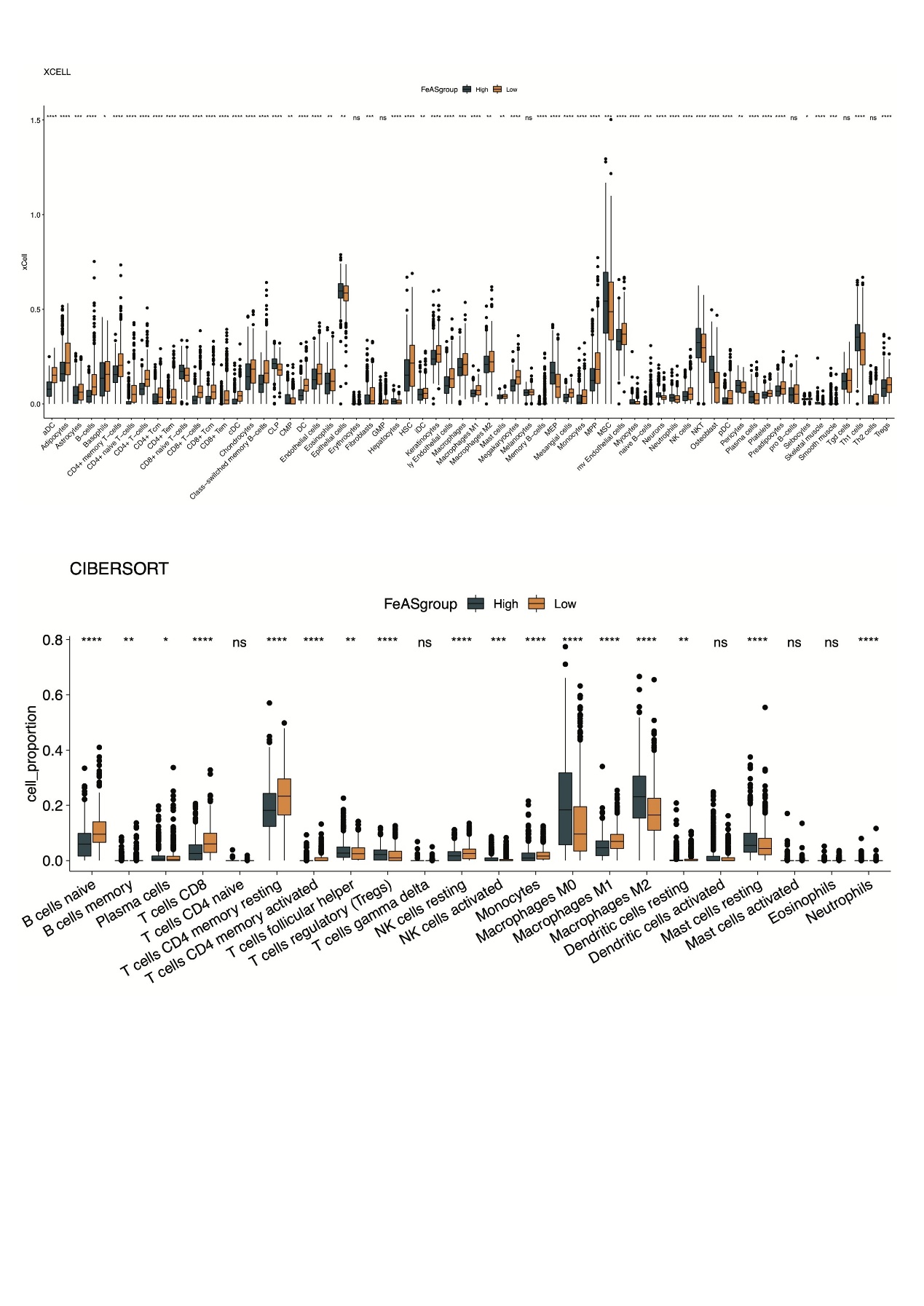
**
