# Supplementary figures and images for: Application of single-cell RNA sequencing analysis of novel breast cancer phenotypes based on the activation of ferroptosis-related genes
Source: Funct Integr Genomics. 2023 May 22;23(2):173. doi: 10.1007/s10142-023-01086-0 (PMC10203036; doi:10.1007/s10142-023-01086-0)

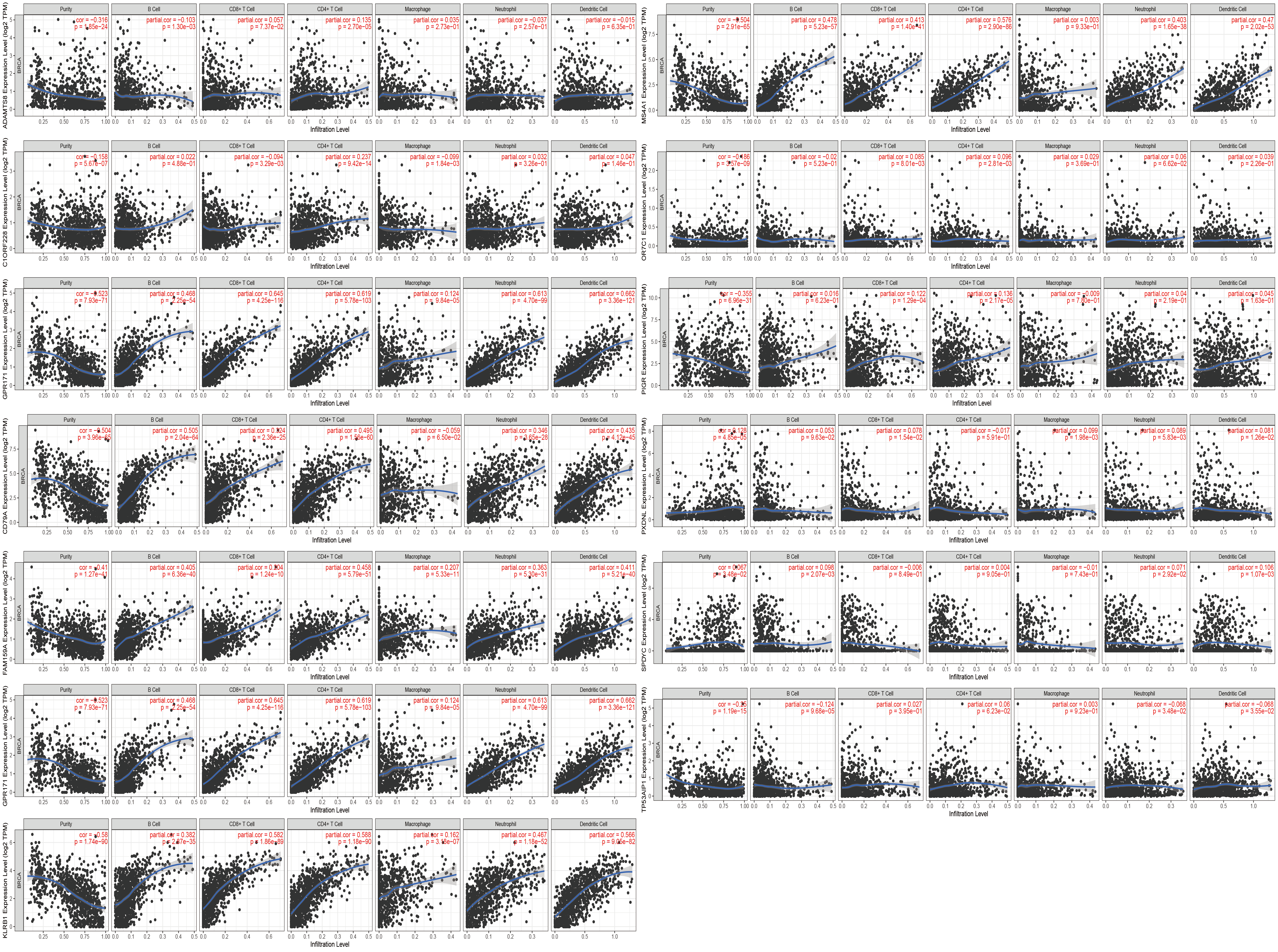

Supplement: Supplementary file 2 — (PNG 3113 kb) [file 10142_2023_1086_Fig16_ESM.png]

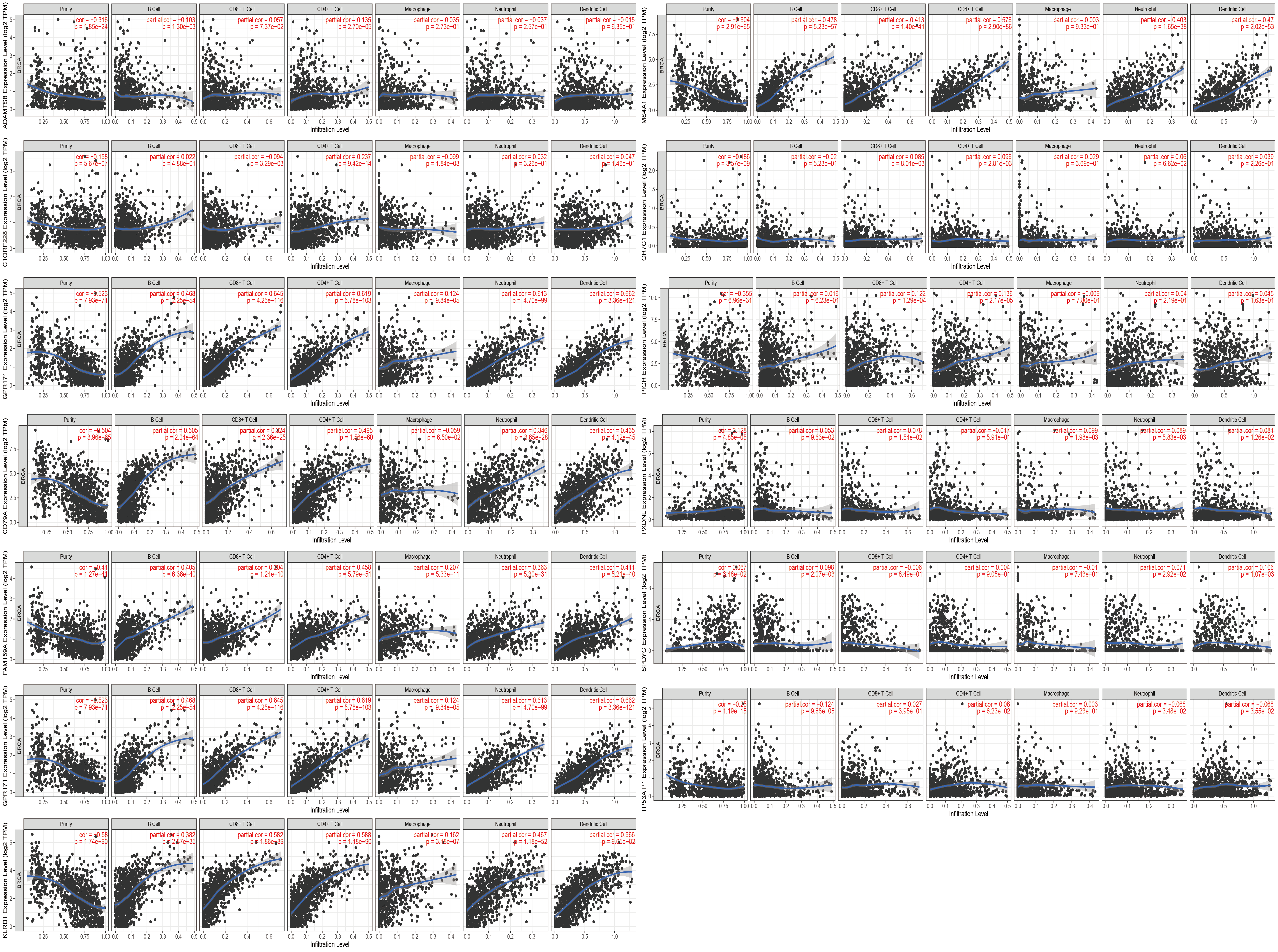

Supplement: Supplementary file 3 — High resolution image (TIF 3078 kb) [file 10142_2023_1086_MOESM2_ESM.tif]
